# Psychometric properties of multicomponent tools designed to assess frailty in older adults: A systematic review

**DOI:** 10.1186/s12877-016-0225-2

**Published:** 2016-02-29

**Authors:** Jennifer L. Sutton, Rebecca L. Gould, Stephanie Daley, Mark C. Coulson, Emma V. Ward, Aine M. Butler, Stephen P. Nunn, Robert J. Howard

**Affiliations:** Department of Old Age Psychiatry, Institute of Psychiatry, Psychology & Neuroscience, King’s College London, Box PO70, De Crespigny Park, London, SE5 8AF UK; Centre for Dementia Studies, Brighton and Sussex Medical School, East Sussex, UK; Department of Psychology, School of Science and Technology, Middlesex University, London, UK; Division of Psychiatry, University College London, London, UK

**Keywords:** Frailty, Assessment, Older adults

## Abstract

**Background:**

Frailty is widely recognised as a distinct multifactorial clinical syndrome that implies vulnerability. The links between frailty and adverse outcomes such as death and institutionalisation have been widely evidenced. There is currently no gold standard frailty assessment tool; optimizing the assessment of frailty in older people therefore remains a research priority. The objective of this systematic review is to identify existing multi-component frailty assessment tools that were specifically developed to assess frailty in adults aged ≥60 years old and to systematically and critically evaluate the reliability and validity of these tools.

**Methods:**

A systematic literature review was conducted using the standardised COnsensus‐based Standards for the selection of health Measurement INstruments (COSMIN) checklist to assess the methodological quality of included studies.

**Results:**

Five thousand sixty-three studies were identified in total: 73 of which were included for review. 38 multi-component frailty assessment tools were identified: Reliability and validity data were available for 21 % (8/38) of tools. Only 5 % (2/38) of the frailty assessment tools had evidence of reliability and validity that was within statistically significant parameters and of fair-excellent methodological quality (the Frailty Index-Comprehensive Geriatric Assessment [FI-CGA] and the Tilburg Frailty Indicator [TFI]).

**Conclusions:**

The TFI has the most robust evidence of reliability and validity and has been the most extensively examined in terms of psychometric properties. However, there is insufficient evidence at present to determine the best tool for use in research and clinical practice. Further in-depth evaluation of the psychometric properties of these tools is required before they can fulfil the criteria for a gold standard assessment tool.

**Electronic supplementary material:**

The online version of this article (doi:10.1186/s12877-016-0225-2) contains supplementary material, which is available to authorized users.

## Background

It is estimated that between the years 2000 and 2050, the percentage of the world’s population over 60 years old will double from 11 to 22 % [1]. Frailty is considered one of the most complex and important issues associated with human ageing, with significant implications for both patient outcomes and healthcare service utilisation. The links between frailty and increased risk of adverse outcomes such as falls, loss of functional independence, decreased quality of life, institutionalisation and mortality have been clearly evidenced [2–7].

A recent systematic review of frailty prevalence worldwide concluded that 10.7 % of community dwelling adults aged ≥65 years were frail and 41.6 % pre-frail [8]. It was noted that prevalence figures varied substantially between studies (ranging from 4.0 to 59.1 %), with studies applying a physical phenotypical definition of frailty consistently reporting lower prevalence rates than those utilising a broader definition of frailty which included psychosocial domains [8]. This highlights the potential disparities in the identification of frailty depending on the definition of frailty applied.

The issue of identifying frailty is compounded by the fact that there is currently no universally accepted operational definition of frailty. A recent Delphi methods based consensus statement on frailty concluded that additional research into clinical and laboratory biomarkers of frailty is needed before an operational definition of frailty can be achieved [9]. However, expert agreement was reached on the basic theoretical underpinnings of frailty; the results of which were reflective of the defining characteristics of frailty for which there is a consensus in the literature. It is widely recognised that frailty is a distinct multifactorial clinical syndrome or state that is separate from, but often associated with, disease and disability [9–11]. Frailty is considered to be a dynamic, non-linear process characterised by decreased reserves and resistance resulting in poor maintenance of physiological homeostasis [10–12]. The dynamic nature of the frailty syndrome gives rise to the potential for preventative and restorative interventions.

Many models have been suggested to conceptualise frailty, however, at present there is no gold standard. The two models which have the largest evidence-base and are the most widely accepted are the Cardiovascular Health Study (CHS) Phenotype Model [13] and the Canadian Study of Health and Ageing (CSHA) Cumulative Deficit Model [14]. The CHS Phenotype Model [13] establishes a frailty phenotype with 5 variables (involuntary weight loss, self-reported exhaustion, slow gait speed, weak grip strength and self-reported sedentary behaviour), whereas the CSHA Cumulative Deficit Model [14] measures frailty via an index of age-related deficits including diseases and disabilities.

A wide variety of tools to screen for, diagnose and measure frailty have been developed based on models of frailty. However, at present no existing assessment tool is considered to be of a gold standard. In view of the predicted rise in the world’s older adult population, the prevalence of frailty in this population, the evidenced links between frailty and adverse outcomes, and the potential for preventative and restorative interventions, the accurate assessment of frailty remains a significant clinical and research priority.

Six systematic reviews regarding the assessment of frailty have been published to date [15–20]. One review focused on the identification of frailty assessment tools [15]. Two reviews focused on the diagnostic test accuracy of frailty assessment tools; one reviewed the accuracy of simple measures to assess frailty [16] and one reviewed the sensitivity, specificity and predictive validity of instruments based on major theoretical views of frailty [17]. A further review examined the criterion validity, construct validity and responsiveness specifically of Frailty Indexes [18]. These reviews focused on the appraisal of a specific subset of frailty assessment tools and did not examine all aspects of validity or explore the reliability of the tools identified. Only two reviews have reported an evaluation of both the reliability and validity of frailty assessment tools [19, 20]; the literature searches for which were completed in February 2010 and May 2011, respectively. Given the current vast expansion of the frailty literature, an updated review in this area is justified. The evaluation of psychometric properties was not the sole focus in either review [19, 20]. An in-depth evaluation of all available reliability and validity data for existing frailty assessment tools; including an assessment of both the methodological quality of the evidence presented and the statistical significance of the results has not been completed. Further, both of these earlier reviews included studies which reported the assessment of frailty via tools that were developed to assess alternative constructs such as disability rather than frailty per se. Tools that have been developed to assess alternate constructs will be based on alternative conceptual models and frameworks that do not represent all aspects of frailty; resulting in limited construct validity when applied to the measurement of frailty. Also, where a tool has been developed to measure a concept that is distinct from but linked to frailty, such as disability, there is a significant chance of confounding of the assessments results, leading to the inaccurate assessment and diagnosis of frailty based on disability factors alone. The inclusion of such tools in a review limits the conclusions that can be drawn in specific reference to the assessment of frailty. One review also included studies involving single-component assessment tools such as grip strength as a single measure [19]. Given the multifactorial and complex nature of the frailty syndrome, a tool to assess frailty should be multicomponent to capture this multifactorial complexity and grounded within a robust evidence-based model of frailty. Tools originally created to assess an alternative concept but later applied to frailty assessment suggest a lack of theoretical robustness, as does the application of a single-component assessment tool to assess a multifactorial clinical syndrome. Consequently, the aims of this review were to: Systematically and critically evaluate the available evidence concerning the reliability and validity of multi-component frailty assessment tools that were specifically developed to assess frailty in older adult populations; establishing the tool with the best evidence to support its use in both research and clinical settings.

## Methods

### Search strategy

The following databases were searched on March 30 2015: Medline (1946–present), PsychINFO (1806–present), Embase (1947–present) and the Cochrane Central Register of Controlled Trials. The search strategy used was: frailty AND (older OR elder* OR geriatr*) AND (measure* OR assess*). The reference lists of previous reviews concerning the measurement of frailty were also searched manually [15–20].

### Selection criteria

Studies were selected for inclusion for review if they met the following criteria:

- Study participants were aged ≥60 years old.
- The study described a multi-component tool (defined as a tool that assesses ≥2 indicators of frailty. Single-component tools were excluded due to the multifactorial and complex nature of the frailty syndrome).
- The study described a tool that was specifically developed to assess frailty (tools which were developed for alternative purposes and then applied to measure frailty were excluded as they do not exclusively assess frailty, but may assess related constructs such as disability resulting in a potentially invalid assessment of frailty and misdiagnosis).
- The main purpose of the study was the development and/or evaluation of the reliability and validity of a multi-component tool to assess frailty.
- The study applied the original version of a multi-component tool to assess frailty (studies citing modified versions were excluded as reliability and validity data relate to the modified tool only and reviewing all modified versions was beyond the scope of this review due to the large number of modified tools identified in the literature).
- The study reported quantitative data (the study must have reported inferential validation, studies reporting descriptive data alone were excluded).
- Studies were available in English or were translated wherever possible.

Studies were screened and selected for inclusion by JLS.

### Assessment of the methodological quality of studies and data extraction

The COnsensus‐based Standards for the selection of health Measurement INstruments (COSMIN) checklist is a standardized tool for assessing the methodological quality of studies examining the measurement properties of health-related instruments [21–23]. It assesses measurement properties in a number of domains: Internal Consistency (the degree of the inter-relatedness among items), Reliability (the proportion of the total variance in measurements due to “true” differences among patients), Measurement Error (the systematic and random error of a patient’s score that is not attributed to true changes in the construct to be measured), Content Validity (the degree to which the content of an instrument is an adequate reflection of the construct to be measured), Construct Validity (the degree to which the scores of an instrument are consistent with hypotheses based on the assumption that the instrument validly measures the construct to be measured), Criterion Validity (the degree to which the scores of an instrument are an adequate reflection of a “gold standard”) and Responsiveness (the ability of an instrument to detect change over time in the construct to be measured) [22]. A ‘’gold standard” measurement instrument is defined in the context of the COSMIN checklist as a valid and reliable instrument that has been widely accepted as a gold standard by experts in the field of its application [21–23].

Structural Validity (the degree to which the scores of an instrument are an adequate reflection of the performance of the dimensionality of the construct to be measured), Hypothesis Testing (item construct validity; the formulation of a hypothesis a priori with regard to correlations between the scores on the instrument and other variables e.g. with regard to internal relationships or relationships with scores on other instruments) and Cross Cultural Validity (the degree to which the performance of the items on a translated or culturally adapted instrument are an adequate reflection of the performance of the items of the original instrument) are assessed as part of Construct Validity [22].

With respect to scoring, each item in the COSMIN checklist is rated as ‘excellent’, ‘good’, ‘fair’, or ‘poor’ quality [21–23]. A rating of ‘excellent’ indicates that the evidence provided for that measurement property is adequate [21]. A rating of ‘good’ indicates that the evidence provided can be assumed to be adequate (although all relevant information may not be reported) [21]. Finally, ratings of ‘fair’ and ‘poor’ indicate that the evidence provided is questionable and inadequate, respectively [21].

The COSMIN checklist was applied to each study and data were extracted by two independent, blind raters (JLS, RLG, MCC, AMB, EVW, SD, SPN). Any disagreements were resolved through discussion. Data were then extracted regarding the methods and outcomes of the statistical analyses employed in each study to assess the identified measurement properties of each assessment tool. The outcomes of the statistical analyses employed by each study were compared to the accepted statistical parameters of significance for said test as identified in medical statistics literature (see Additional file 1 footnote). This allowed for the identification of statistically significant evidence of measurement properties testing.

### Reporting

This review followed the PRISMA standards [24] for reporting of systematic reviews (see Additional file 2).

## Results

### Literature search and inclusion for review

**Fig. 1:**
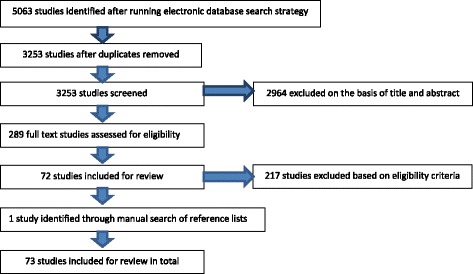
Process of study selection

Five thousand sixty-three studies were identified in total, 73 of which were included for review following assessment against inclusion criteria (see Fig. 1) [2, 13, 25–95].

### Study characteristics

Thirty-eight multi-component frailty assessment tools were examined in 73 studies. The most frequently examined tool with respect to psychometric properties was the Groningen Frailty Indicator (GFI), which was assessed in 11 studies [27, 49, 54–62]. The Tilburg Frailty Indicator (TFI) was also frequently examined, with 9 studies included for review [55, 57, 88–94]. Psychometric properties were assessed in 1 study only for 22/38 tools [25, 28–30, 34, 38, 44, 48, 51–53, 63, 66–68, 71–73, 79, 86, 87, 95]. Prospective Cohort was the most frequently observed study design (22/73 studies) [2, 13, 25–29, 31–33, 47, 48, 53, 55, 60, 66, 67, 72, 80, 81, 86, 95]. In 54/73 studies the cohort was exclusively community-based [2, 13, 25, 26, 29, 31, 33, 34, 38–40, 45, 46, 48–50, 52–56, 59, 63–67, 70, 71, 73–95]. The country from which participants were most commonly sampled was The Netherlands (26/73 studies) [39, 40, 44, 49, 50, 54–63, 65, 72, 74–81, 89–93]. Follow-up data were available for 51/73 studies; follow-up periods varied significantly with the shortest being 1 month [35] and the longest 348 months [33]. Data regarding the mean age of participants were available in 55/73 studies; the overall mean age of the participants as calculated by pooling the mean ages from these 55 studies was 77.0 years [2, 25, 27, 28, 30, 32, 33, 35–42, 44, 47–50, 52–59, 61–65, 67–69, 72, 74–76, 79–84, 86–94]. A full outline of study characteristics is provided in Additional file 3.

### Methodological quality of studies

The results of the COSMIN checklist are summarised in Table 1. 38/73 studies included for review had at least one area of methodological quality rated as ‘poor’, indicating inadequate quality [27, 29–35, 38–41, 43, 44, 46, 49, 51–57, 60, 61, 67–70, 74, 75, 78, 81, 82, 85, 86, 90, 94]. The measurement property that received the highest number of poor ratings across all studies was Criterion Validity (23/44 total ‘poor’ ratings). 52/73 studies had at least one area of methodological quality rated as ‘fair’, indicating questionable methodological quality [2, 13, 25, 26, 28, 30, 31, 34–39, 42, 44–50, 54–56, 60–67, 71–81, 83, 85–87, 91–93, 95]. The measurement property that received the highest number of ‘fair’ ratings was Hypothesis Testing (50/64 total ‘fair’ ratings). 2/73 studies had one area of methodological quality scored as ‘good’, indicating presumably adequate methodological quality [57, 58]. All ratings of ‘good’ quality were awarded for Hypothesis Testing. 6/73 studies had one area of methodological quality scored as ‘excellent’, indicating adequate methodological quality [25, 31, 54, 67, 74, 79, 84]. All ratings of ‘excellent’ were awarded for Content Validity.

**Table 1:** Results of COnsensus‐based Standards for the selection of health Measurement Instruments (COSMIN) Checklist

Frailty assessment tool	Study reference	Internal consistency	Reliability	Measurement error	Content validity	Structural validity	Hypotheses testing	Cross-cultural validity	Criterion validity	Responsiveness	Comments
9-Item Frailty Measure	Ravaglia et al. [25]	0	0	0	4	-	2	0	0	0	Structural Validity: Not rated according to COSMIN guidance as the tool is based on a formative model.
Brief Clinical Instrument to Classify Frailty	Rockwood et al. [26]	0	0	0	0	0	2	0	-	0	Criterion Validity: Study reports assessment of criterion validity however this is rated as part of construct validity (hypothesis testing) according to COSMIN guidance.
	Kenig et al. [27]	0	0	0	0	0	1	0	0	0	Hypothesis Testing: Important methodological flaws in the design of the study noted; diagnosis of frailty via the detection of deficits in two or more domains of the Geriatric Assessment (GA) has limited theoretical grounding. No reliability or validity data for GA in this context.
Brief Frailty Index	Freiheit et al. [28]	0	0	0	2	0	2	0	0	0	
British Frailty Index	Kamaruzzaman et al. [29]	-	0	0	0	0	0	0	1	0	Internal Consistency: Not rated according to COSMIN guidance as the tool is based on a formative model. Criterion Validity: The criterion employed cannot be considered a reasonable gold standard. Correlations or AUC of ROC not calculated.
Care Partners-Frailty Index-Comprehensive Geriatric Assessment (CP-FI-CGA)	Goldstein et al. [30]	0	0	0	2	0	2	0	1	0	Criterion Validity: The criterion employed cannot be considered as a reasonable gold standard.
Clinical Frailty Scale	Rockwood et al. [31]	0	1	0	4	0	2	0	1	0	Reliability: Tool not administered by independent raters when assessing inter-rater reliability. Criterion Validity: The criterion employed cannot be considered as a reasonable gold standard.
	Rockwood et al. [32]	0	0	0	0	0	1	0	1	0	Criterion Validity: The criterion employed cannot be considered as a reasonable gold standard. Criterion Validity and Hypothesis Testing: Important methodological flaws in the design of the study noted; measurement properties of comparator instrument (Phenotype of frailty-Referred to as Frail-CHS) significantly altered from original. No reliability or validity data for amended version.
	Mitiniski et al. [33]	0	0	0	0	0	1	0	0	0	Construct Validity: Important methodological flaws in the design of the study noted; measurement properties of comparator instrument (Phenotype of Frailty) significantly altered from original. No reliability or validity data for amended version.
Clinical Global Impression of Change in Physical Frailty	Studenski et al. [34]	0	1	0	2	0	0	0	0	0	Reliability: Inter-rater reliability assessed using case scenarios. Small sample size (n = 24) for pilot testing. Likely selection bias in the focus group; all patients and carers selected by first author (all carers were female). Likely selection bias for participants of pilot test as the testing physicians chose two patients who they deemed to be frail to be tested.
Comprehensive Assessment of Frailty (CAF)	Sundermann et al. [35]	0	0	0	1	-	2	0	0	0	Content validity: Some aspects of content validity explored however there was limited assessment of whether all items are relevant for the study population and limited information regarding the theoretical foundation of the tool. Structural validity: Not rated according to COSMIN guidance as the tool is based on a formative model.
	Sundermann et al. [36]	0	0	0	0	0	2	0	0	0	
	Sundermann et al. [37]	0	0	0	0	0	2	0	0	0	
Continuous Composite Measure of Frailty	Buchman et al. [38]	0	0	0	2	0	2	0	1	1	Criterion Validity: The criterion employed cannot be considered as a reasonable gold standard. Measurement properties of comparator instrument (Phenotype of Frailty) significantly altered from original. No reliability or validity data for amended version. Responsiveness: The time interval between measurements was not adequately described.
EASY-Care Two-step Older persons Screening (Easycare TOS)	Van Kempen et al. [39]	0	1	0	0	0	2	0	1	0	Reliability: Small sample size (*n* = 19) for reliability calculations. Criterion Validity: The criterion employed cannot be considered as a reasonable gold standard.
	Van Kempen et al. [40]	0	0	0	1	0	0	0	0	0	Content Validity: An assessment of whether all items are relevant for the purpose of the measurement instrument was competed however limited information available regarding other aspects of content validity.
Edmonton Frail Scale	Rolfson et al. [41]	-	1	0	0	0	0	0	0	0	Internal Consistency: Not rated according to COSMIN guidance as the tool is based on a formative model. Reliability: Small sub sample size (*n* = 18) for reliability calculations.
	Haley et al. [42]	0	0	0	0	0	2	0	0	0	
	Graham et al. [43]	0	0	0	0	0	1	0	0	0	Hypothesis Testing: Important methodological flaws in the design of the study noted; tertile split was performed for reasons of sample size equality and not theoretically or empirically justified.
Evaluative Index for Physical Frailty	De Vries et al. [44]	0	1	0	2	0	1	0	0	0	Reliability and Hypothesis testing: Small sample (*n* = 24)
Frailty Index-Comprehensive Geriatric Assessment (FI-CGA)	Jones et al. [45]	0	2	0	2	0	2	0	0	0	
	Jones et al. [46]	0	0	0	0	0	2	0	1	0	Criterion Validity: The criterion employed cannot be considered as a reasonable gold standard.
	Pilotto et al. [47]	0	0	0	0	0	2	0	0	0	
FORECAST	Sundermann et al. [35]	0	0	0	0	0	2	0	0	0	
	Sundermann et al. [36]	0	0	0	0	0	2	0	0	0	
Frailty Index	Mitnitski et al. [48]	0	0	0	0	0	2	0	0	0	
Frailty Index based on Primary Care Data.	Drubbel et al. [49]	0	0	0	2	0	2	0	1	0	Criterion Validity: The criterion employed cannot be considered as a reasonable gold standard.
	Drubbel et al. [50]	0	0	0	2	0	2	0	0	0	
Frailty Index for Elders (FIFE)	Tocchi et al. [51]	-	0	0	1	-	0	0	0	0	Internal Consistency & Structural Validity: Not rated according to COSMIN guidance as the tool is based on a formative model. Content Validity: Important methodological flaws in the design of the study noted; during item generation process potential variables excluded solely on the basis of information available in the parent data set.
Frail Non- Disabled Instrument (FiND)	Cesari et al. [52]	0	0	0	1	0	1	0	1	0	Content Validity, Hypothesis Testing and Criterion Validity: Important methodological flaws in the design of the study noted; Analysis of agreement between FiND and Phenotype of Frailty is flawed as FiND includes 2/5 of the Phenotype of Frailty items. This significantly affects the interpretation of the data.
Frailty Screening Tool	Doba et al. [53]	0	0	0	1	-	1	0	0	0	Structural Validity: Not rated according to COSMIN guidance as the tool is based on a formative model. Content validity & Hypothesis Testing: Important methodological flaws in the design of the study noted; potential selection bias due to the exclusion of those older adults who had chronic comorbid illness. Potential underrepresentation of frailest adults due to the exclusion of participants from analysis whom were unable to engage in a final assessment. No clarity regarding the definition of ’cognitive change’ item.
Groningen Frailty Indicator (GFI)	Bielderman et al. [54]	-	0	0	4	-	2	0	1	0	Internal Consistency and Structural Validity: Not rated according to COSMIN guidance as the tool is based on a formative model. Criterion Validity: The criterion employed cannot be considered as a reasonable gold standard.
	Daniels et al. [55]	0	0	0	0	0	2	0	1	2	Criterion Validity: The criterion employed cannot be considered as a reasonable gold standard.
	Drubbel et al. [49]	0	0	0	0	0	2	0	1	0	Criterion Validity: The criterion employed cannot be considered as a reasonable gold standard.
	Hoogendijk et al. [56]	0	0	0	0	0	2	0	1	0	Criterion Validity: The criterion employed cannot be considered as a reasonable gold standard.
	Kenig et al. [27]	0	0	0	0	0	1	0	0	0	Hypothesis Testing: Important methodological flaws in the design of the study noted; diagnosis of frailty via the detection of deficits in two or more domains of the Geriatric Assessment has limited theoretical grounding. No reliability or validity data for GA in this context.
	Metzelthin et al. [57]	-	0	0	0	-	3	0	1	0	Internal Consistency and Structural Validity: Not rated according to COSMIN guidance as the tool is based on a formative model. Criterion Validity: The criterion employed cannot be considered as a reasonable gold standard.
	Peters et al. [58]	-	0	0	0	-	3	0	0	0	Internal Consistency and Structural Validity: Not rated according to COSMIN guidance as the tool is based on a formative model.
	Schuurmans et al. [59]	0	0	0	0	0	2	0	0	0	
	Smets et al. [60]	0	0	0	0	0	2	0	1	0	Criterion Validity: The criterion employed cannot be considered as a reasonable gold standard.
	Steverink et al. [61]	-	1	0	1	0	2	0	0	0	Internal Consistency: Not rated according to COSMIN guidance as the tool is based on a formative model. Reliability: Limited information regarding basic inter-rater reliability calculations given. No further reliability calculations completed. Content Validity: Limited assessment of whether all items are relevant for the study population. Limited information due to source being a poster presentation abstract.
	Tegels et al. [62]	0	0	0	0	0	2	0	0	0	
Guilley Frailty Instrument	Guilley et al. [63]	0	0	0	0	0	2	0	0	0	
Inactivity and Weight Loss	Chin et al. [64]	0	0	0	2	0	2	0	0	0	
	Chin et al. [65]	0	0	0	0	0	2	0	0	0	
INTER-FRAIL Study Questionnaire	De Bari et al. [66]	0	0	0	2	0	2	0	0	0	
KLoSHA Frailty Index	Jung et al. [67]	0	0	0	4	-	2	0	1	0	Structural Validity: Not rated according to COSMIN guidance as the tool is based on a formative model. Criterion Validity: The criterion employed cannot be considered as a reasonable gold standard.
Marigliano–Cacciafesta Polypathological Scale	Amici et al. [68]	0	0	0	0	0	1	0	1	0	Hypothesis testing and Criterion Validity: Important methodological flaws in the design of the study noted; analysis consists purely of correlations with limited theoretical justification. Criterion Validity: The criterion employed cannot be considered as a reasonable gold standard.
Phenotype of Frailty	Esrund et al. [2]	0	0	0	0	0	2	0	0	0	
	Fried et al. [13]	0	0	0	0	0	2	0	0	0	
	Kenig et al. [27]	0	0	0	0	0	1	0	0	0	Hypothesis Testing: Important methodological flaws in the design of the study noted; diagnosis of frailty via the detection of deficits in two or more domains of the Geriatric Assessment has limited theoretical grounding. No reliability or validity data for GA in this context.
	Kim et al. [69]	0	0	0	0	0	0	0	1	0	Criterion Validity: The criterion employed cannot be considered as a reasonable gold standard. No calculations of sensitivity and specificity.
	Kulminski et al. [70]	0	0	0	0	0	0	0	1	0	Criterion Validity: The criterion employed cannot be considered as a reasonable gold standard. Scoring changed from ordinal to continuous however only relative risk ratios were compared. No correlations or AUC of ROC calculated.
Predictive Physical Frailty Score	Carriere et al. [71]	0	0	0	0	-	2	0	0	0	Structural Validity: Not rated according to COSMIN guidance as the tool is based on a formative model.
Prognostic Risk Score	Pijpers et al. [72]	0	0	0	0	-	2	0	0	0	Structural Validity: Not rated according to COSMIN guidance as the tool is based on a formative model.
Self-Report Screening Tool for Frailty	De Souto Barreto et al. [73]	0	0	0	0	0	2	0	0	0	
SHARE Frailty Instrument	Romero-Ortuno et al. [74]	0	0	0	4	-	2	0	1	0	Structural Validity: Not rated according to COSMIN guidance as the tool is based on a formative model. Criterion Validity: The criterion employed cannot be considered as a reasonable gold standard.
	Romero-Ortuno et al. [75]	0	0	0	0	0	2	0	1	0	Criterion Validity: The criterion employed cannot be considered as a reasonable gold standard.
	Romero-Ortuno et al. [76]	0	0	0	0	0	2	0	0	0	
	Romero-Ortuno et al. [77]	0	0	0	0	0	2	0	0	0	
	Romero-Ortuno et al. [78]	0	0	0	0	0	2	0	1	0	Criterion Validity: The criterion employed cannot be considered as a reasonable gold standard.
SHARE Frailty Instrument 75+ (SHARE-FI75+)	Romero-Ortuno et al. [79]	-	0	0	4	-	2	0	0	0	Internal Consistency & Structural Validity: Not rated according to COSMIN guidance as the tool is based on a formative model.
SOF Frailty Criteria	Bilotta et al. [80]	0	0	0	0	0	2	0	0	0	
	Ensrud et al. [81]	0	0	0	0	0	2	0	1	0	Criterion Validity: The criterion employed cannot be considered as a reasonable gold standard.
Strawbridge Frailty Measure	Strawbridge et al. [82]	0	0	0	1	0	0	0	0	0	Content validity: No assessment of whether all items are relevant for the target population and no assessment of whether all items together comprehensively reflect the measurement of frailty.
	Matthews et al. [83]	0	0	0	0	0	2	0	0	0	
The Comprehensive Frailty Assessment Instrument	De Witte et al. [84]	-	0	0	4	-	0	0	0	0	Internal Consistency & Structural Validity: Not rated according to COSMIN guidance as the tool is based on a formative model.
	De Witte et al. [85]	-	0	0	0	0	2	0	1	0	Internal consistency: Not rated according to COSMIN guidance as the tool is based on a formative model. Criterion Validity: The criterion employed cannot be considered as a reasonable gold standard.
The Frailty Trait Scale	Garcia-Garcia et al. [86]	0	0	0	2	0	2	0	1	0	Criterion Validity: The criterion employed cannot be considered as a reasonable gold standard.
The FRAIL Scale	Lopez et al. [87]	0	0	0	0	0	2	0	0	0	
Tilburg Frailty Indicator (TFI)	Andreasen et al. [88]	0	0	0	0	0	0	1	0	0	Cross-Cultural Validity: Sample size less than 5 times the number of items included on the scale (5* 15 = 75, actual sample size included; 34)
	Daniels et al. [55]	0	0	0	0	0	2	0	1	2	Criterion Validity: The criterion employed cannot be considered as a reasonable gold standard.
	Gobbens & van Assen [89]	0	0	0	0	0	2	0	0	0	
	Gobbens et al. [90]	0	0	0	1	0	0	0	0	0	Content Validity: An assessment of whether all items are relevant for the purpose of the measurement instrument was competed however there was limited information regarding other aspects of content validity.
	Gobbens et al. [91]	0	2	0	2	0	2	0	0	0	
	Gobbens et al. [92]	0	0	0	0	0	2	0	0	0	
	Gobbens et al. [93]	0	0	0	0	0	2	0	0	0	
	Metzelthin et al. [57]	-	0	0	0	-	3	0	1	0	Internal Consistency and Structural Validity: Not rated according to COSMIN guidance as the tool is based on a formative model. Criterion Validity: The criterion employed cannot be considered as a reasonable gold standard.
	Uchmanowicz et al. [94]	-	0	0	0	0	0	1	0	0	Internal Consistency: Not rated according to COSMIN guidance as the tool is based on a formative model. Cross Cultural Validity: Multiple-group confirmatory factor analysis not performed.
WHIOS Multicomponent Measure	Woods et al. [95]	0	0	0	0	0	2	0	0	0	

Key: 4: Excellent, 3: Good, 2: Fair, 1: Poor, 0: No information, − : Not rated

*AUC* Area Under Curve, *ROC* Receiver Operating Curve

### Psychometric properties of the multi-component frailty assessment tools

Table 1 provides an overview of the measurement properties of each multi-component frailty assessment tool. The tools that have been examined the most with respect to psychometric domains were the TFI and GFI. The TFI had 8 of the possible 9 domains explored (the exception being Measurement Error) [55, 57, 88–94]. The GFI had 7/9 domains examined (the exceptions being Measurement Error and Cross Cultural Validity) [27, 49, 54–62]. The tools that were examined the least with respect to psychometric domains were Frailty predicts death One yeaR after Elective Cardiac Surgery Test (FORECAST) [36, 37], Guilley Frailty Instrument [63], Self-Report Screening Tool for Frailty [73], The Fatigue Resistance Ambulation Illnesses Loss of Weight (FRAIL) Scale [87] and Women's Health Initiative Observational Study (WHIOS) Multicomponent Measure [95]. Each of these tools had only one element of Construct Validity (Hypothesis Testing) explored.

Overall Internal Consistency was assessed in 7/38 tools [29, 41, 51, 54, 57, 58, 61, 79, 84, 85, 94]; Internal Consistency was determined via Cronbach α calculations for 6/7 tools, the scores of which ranged from 0.62 for the Edmonton Frail Scale (EFS) [42] to 0.81 for The Comprehensive Frailty Assessment Instrument [84]. Reliability was assessed in 8/38 tools [31, 34, 39, 41, 44, 45, 61, 91]. Inter-rater reliability was assessed for 8/38 tools [31, 34, 39, 41, 44, 45, 61, 91] and was most commonly assessed using Cohen’s Kappa calculations, the scores of which ranged from 0.63 for the Easycare- Two-step Older persons Screening (EASY-Care TOS) [39] to 0.72 for the Evaluative Index for Physical Frailty (EIPF) [44]. Intra-rater reliability was assessed for the EIPF only using Cohen’s Kappa calculations and Intraclass Correlation Coefficient calculations [44]. Test-retest reliability was assessed for the TFI only using Pearson Correlation Coefficient calculations [91]. Measurement Error was not assessed for any tool.

Construct Validity was the most widely evaluated measurement property, and was assessed in 36/38 tools [2, 13, 25–28, 30–33, 35–95]. The Clinical Global Impression of Change in Physical Frailty [34] and the British Frailty Index (BFI) [29] were the only tools for which Construct Validity was not assessed. Structural Validity was assessed in 12/38 tools [25, 35, 51, 53, 54, 57, 58, 67, 71, 72, 74, 79, 84]. Exploratory and Confirmatory Factor Analysis were the most common statistical methods employed to determine structural validity. Hypothesis Testing was assessed in 33/38 tools [2, 13, 25–28, 30–33, 35–39, 42–50, 52–68, 71–81, 83, 85–87, 89, 91–93, 95]. Hazard and Odds Ratios were the most frequently employed method of statistical analysis used to establish predictive validity as part of Hypothesis Testing. Cross Cultural Validity was assessed in one tool; the TFI [88, 94]. Content Validity was assessed in 28/38 tools [25, 28, 30, 31, 34, 35, 38, 40, 44, 45, 49–54, 61, 66, 67, 74, 79, 82, 84, 86, 90, 91]. Criterion Validity was assessed in 18/38 tools [26, 29–32, 38, 39, 46, 49, 52, 54–57, 60, 67–70, 74, 75, 78, 81, 85, 86]. Receiver Operating Characteristic curve analysis was the most frequently employed method of statistical analysis to determine Criterion Validity. Responsiveness was assessed in 2/38 tool; the GFI and TFI [55]. Additional file 1 provides an overview of the statistical analysis employed in each study to assess the identified measurement properties.

Table 2 summarises the measurement properties evaluated for each tool for which the supporting evidence was within statistically significant parameters and the evidence was rated as ‘fair’, ‘good’ or ‘excellent’ according to the COSMIN checklist. Evidence of Internal Consistency and Structural Validity was excluded following COSMIN guidance as items of a measurement tool do not need to be correlated when a tool is based on a formative model [21–23].

**Table 2:** A summary of the measurement properties of multicomponent frailty assessment tools with evidence of reliability and validity that was within statistical significant parameters and of fair to excellent quality

Frailty assessment tool	Internal consistency	Reliability	Measurement error	Content validity	Structural validity	Hypothesis testing	Cross cultural validity	Criterion validity	Responsiveness
9-Item Frailty Measure [25]				X		X			
Brief Clinical Instrument to Classify Frailty [26, 27]						X			
Brief Frailty Index [28]				X		X			
British Frailty Index [29]									
Care Partners-Frailty Index-Comprehensive Geriatric Assessment [30]				X		X			
Clinical Frailty Scale [31–33]				X		X			
Clinical Global Impression of Change in Physical Frailty [34]				X					
Comprehensive Assessment of Frailty [35–37]						X			
Continuous Composite Measure of Frailty [38]				X		X			
EASY-Care Two-step Older persons Screening [39, 40]						X			
Edmonton Frail Scale [41–43]									
Evaluative Index for Physical Frailty [44]				X					
FI-CGA [45–47]		X		X		X			
FORECAST [35, 36]						X			
Frailty Index [48]						X			
Frailty Index based on Primary Care Data [49, 50]				X		X			
Frailty Index for Elders [51]									
Frail Non-Disabled Instrument [52]									
Frailty Screening Tool [53]									
Groningen Frailty Indicator [27, 49, 54–62]				X		X			X
Guilley Frailty Instrument [63]						X			
Inactivity and Weight Loss [64, 65]				X		X			
INTER-FRAIL Study Questionnaire [66]				X		X			
KLoSHA Frailty Index [67]				X		X			
Marigliano–Cacciafesta Polypathological Scale [68]									
Phenotype of Frailty [2, 13, 27, 69, 70]						X			
Predictive Physical Frailty Score [71]						X			
Prognostic Risk Score [72]						X			
Self-Report Screening Tool for Frailty [73]						X			
SHARE Frailty Instrument [74–78]				X		X			
SHARE Frailty Instrument 75+ [79]				X		X			
SOF Frailty Criteria [80, 81]						X			
Strawbridge Frailty Measure [82, 83]									
The Comprehensive Frailty Assessment Instrument [84, 85]				X		X			
The Frailty Trait Scale [86]				X		X			
The FRAIL Scale [87]						X			
Tilburg Frailty Indicator (TFI) [55, 57, 88–94]		X		X		X			X
WHIOS Multicomponent Measure [95]						X			

In terms of the individual measurement properties that were evaluated, 2/38 frailty assessment tools had Reliability data within statistically significant parameters of fair-excellent quality; the FI-CGA [45–47] and TFI [45, 91]. 18/38 tools had Content Validity of fair-excellent quality within statistically significant parameters [25, 26, 30–38, 44–47, 49, 50, 54–62, 64–67, 74–79, 84–86, 88–94]. 30/38 tools had evidence for Hypothesis Testing [2, 13, 25–28, 30–32, 35–39, 42, 45–50, 54–67, 71–81, 83, 85–87, 89, 91–93, 95] and 2/38 had evidence of Responsiveness; the GFI and TFI [55].

The TFI and the FI-CGA were the only tools which had both reliability and validity data within statistically significant parameters of fair-excellent quality [45–47, 55, 57, 88–94]. The TFI had acceptable evidence of psychometric testing for 4 measurement domains; Reliability, Content Validity, Hypothesis Testing and Responsiveness. The FI-CGA had acceptable evidence of psychometric testing for 3 measurement domains; Reliability, Content Validity and Hypothesis Testing. The following tools were found to have no reliability or validity evidence of fair-excellent quality within statistically significant parameters; BFI [29], EFS [41, 42], Frailty Index for Elders [51], Frail Non-Disabled Instrument [52], Frailty Screening Tool [53], Marigliano–Cacciafesta Polypathological Scale [68] and Strawbridge Frailty Measure [82, 83].

## Discussion

To the authors’ knowledge this is the first review of the overall reliability and validity of multi-component frailty assessment tools that were specifically developed to assess frailty in older adult populations. This review presents a comprehensive list of multi-component frailty assessment tools for which there are published psychometric data.

Whilst 73 papers met the inclusion criteria for review, many more were excluded as they directly or indirectly reported on the psychometric evaluation of an amended version of an established frailty assessment tool. This was predominantly observed in relation to the CHS Phenotype Model [13] and the CSHA Cumulative Deficit Model [14], where modified versions of Fried’s Phenotype of Frailty tool and Mitinski’s Frailty Index were applied. While evidence from such studies supports the robustness of these models to conceptualise frailty, it does not provide evidence for the reliability or validity of the original assessment tool. This application of non-standardised versions of frailty assessment tools within frailty research significantly limits conclusions that can be drawn regarding reliability and validity. It is notable that the CSHA Cumulative Deficit Model is not prescriptive regarding the exact age-related deficits to be included in a Frailty Index, nor the exact number of deficits [14]. A wide range of non-standardised Frailty Indexes were identified in the literature, which was outside of the scope of this review to explore; a recent systematic review by Drubbel et al. [18] specifically explored the criterion validity, construct validity and responsiveness of the Frailty Indexes when applied in a community-dwelling older adult population.

It was observed that many of the frailty assessment tools included for review were developed and tested retrospectively using data available from large-scale longitudinal studies or were developed in conjunction with a larger trial; the main aim of which was not the development of a frailty assessment tool. This lack of focused primary research may partly explain why there are limited reliability and validity data of high quality for many of the tools identified.

In summary, the GFI and TFI were the most frequently examined tools with respect to psychometric properties (11 and 9 studies respectively). 22/38 tools identified had only 1 study concerning psychometric properties; this limited evidence-base reduces the generalisability of the results and conclusions that can be drawn.

Health measurement instruments must be both reliable and valid to ensure diagnostic accuracy and consistency in measurement [23]. Of the 38 multi-component frailty assessment tools identified, no tool has been examined in all reliability and validity domains assessed by the COSMIN checklist. The TFI and GFI had the most psychometric domains explored (8/9 and 7/9 domains, respectively). However, not all of this evidence was assessed to be of fair-excellent quality within statistically significant parameters. Only the TFI and FI-CGA had reliability and validity data within statistically significant parameters of fair-excellent quality. The TFI had acceptable evidence of psychometric testing for 4 measurement domains, while the FI-CGA had acceptable evidence of psychometric testing for 3 measurement domains.

### Research and clinical implications

The frailty assessment tool that has been most extensively examined in terms of its psychometric properties and has the most robust evidence supporting its reliability and validity is the TFI. However, for a frailty assessment tool to meet the requirements of a gold standard it must be based on a universally accepted operational definition of frailty and have evidence pertaining to all aspects of the tool’s reliability and validity of high methodological quality [9]. Further research of good-excellent quality is needed, encompassing all aspects of reliability and validity, before the TFI tool can be classified as a gold standard.

The application of a tool without a strong evidence-base of reliability and validity significantly increases the risk of invalid assessment and misdiagnosis of frailty. The consequent implications for research are substantial, including an increased likelihood of the interpretation and reporting of flawed results. The implications for treatment provision and patient outcomes in a clinical setting are also substantial; with potential for decreased recognition of risks for adverse outcomes, inappropriate treatment planning and inappropriate allocation of resources including unsuitable provision of preventative and restorative interventions. Therefore, the scope and quality of reliability and validity evidence must be considered when selecting an assessment tool in both settings. Other key considerations that are important to note when selecting a frailty assessment tool are the interpretability and generalisability of the evidence-base. Evidence of the reliability and validity of an assessment tool relates only to its application within the specific setting and population that it was developed for and validated in. The utility of the tool should also be considered, specifically the appropriateness of the mode of administration in relation to the setting and the time and resource demands associated with the tool.

The development and psychometric evaluation of frailty assessment tools should be the primary focus of research projects to further develop a strong evidence-base. When evaluating existing tools, studies should apply a standardised version where feasible. The consensus on a universally accepted operational definition of frailty should also be a key focus of future frailty research to support the development of a gold standard frailty assessment tool.

### Limitations of the review

The selection of studies for inclusion was completed by the lead author (JLS) only, which increased the potential for selection bias; this risk was minimised by following a comprehensive search strategy and the PRISMA standards for reporting in systematic reviews [24]. Studies examining tools that were not specifically developed to assess frailty were excluded; this resulted in the exclusion of some tools such as the Short Physical Performance Battery [96] and Comprehensive Geriatric Assessment [97] which have been referred to in the frailty literature as tools with potential utility in assessing frailty as part of a wider comprehensive assessment. This limits the scope of this review, but was considered reasonable given the complexity of the frailty syndrome. Studies which directly or indirectly reported on the psychometric evaluation of an amended version of an established frailty assessment tool were also excluded. This again limits the scope of the review but was considered reasonable due to the large number of studies citing modified tools identified in the literature and the large variation in the types of modifications.

The COSMIN checklist has several limitations in its application. When assessing Criterion Validity the COSMIN checklist requires the comparator tool to be of a gold standard. There is currently no gold standard frailty assessment tool. Thus, whilst the majority of studies included for review assessing Criterion Validity compared one frailty assessment tool to another widely-used tool, the COSMIN guidance stipulated that this should be rated as evidence of poor methodological quality in relation to Criterion Validity. The COSMIN guidance does however allow for this relationship between frailty assessment tools to be rated as part of Construct Validity, so the evidence of validity provided by such studies was still represented in the COSMIN scoring system. With regards to the COSMIN scoring system, the overall methodological quality rating per measurement property is obtained by taking the lowest rating of all the items assessed for that property giving a ‘worst counts score’ [21–23]. Occasionally, however, a measurement property scored highly for all items assessed except for one which resulted in a ‘poor’ overall score which did not accurately reflect all the presented evidence. Such a measurement property received the same overall rating as measurement properties that had entirely poor ratings for all items. It was not within the scope of this systematic review to differentiate between such ratings on an item by item basis when reporting results. Whilst this is a limitation, receiving a rating of ‘poor’ for one item is an indication of inadequate methodological quality so it does not impact on the overall quality assessment. The application of the COSMIN checklist; a standardised tool developed specifically to assess the methodological quality of studies examining the measurement properties of health-related instruments remains a strength of this review.

## Conclusions

This review provides an up-to-date comprehensive list of all multi-component frailty assessment tools for which there is published psychometric data. It identifies a large number of multi-component frailty assessment tools in existence; however, the breadth and quality of the psychometric properties of these tools is limited. Only the FI-CGA [45–47] and TFI [54, 56, 86–94] have both reliability and validity data within statistically significant parameters and of fair-excellent quality. However, this should be interpreted with caution as a score of ‘fair’ on the COSMIN checklist means that the evidence is only of questionable quality. At present, the TFI has the most robust evidence-base supporting its reliability and validity in assessing frailty. However, the psychometric properties of the TFI and all other multi-component frailty assessment tools require further in-depth evaluation before they can fulfil the criteria for a gold standard assessment tool, and before definitive conclusions regarding the best tool for use in research and clinical settings can be drawn.

## Additional files

Additional file 1:
**Summary of Key Quantitative Reliability and Validity Data for Multicomponent Frailty Assessment Tools** **[** 98 **–** 100 **].** (DOCX 65 kb) Additional file 2:
**PRISMA Checklist.** (DOCX 23 kb) Additional file 3:
**General Characteristics of Studies.** (DOCX 44 kb)
