# Supplementary material for: Psychometric properties of multicomponent tools designed to assess frailty in older adults: A systematic review
Source: BMC Geriatr. 2016 Feb 29;16:55. doi: 10.1186/s12877-016-0225-2 (PMC4772336; doi:10.1186/s12877-016-0225-2)
Supplement: Additional file 1: — Summary of Key Quantitative Reliability and Validity Data for Multicomponent Frailty Assessment Tools [ 98 – 100 ]. (DOCX 65 kb) [file 12877_2016_225_MOESM1_ESM.docx]

Additional file 1: Summary of Key Quantitative Reliability and Validity Data for Multicomponent Frailty Assessment Tools.

| **Frailty Assessment Tool** | **Reliability &**  **Validity** | **Comments^a^** |
| --- | --- | --- |
| 9-Item Frailty Measure | Validity:   - Content & Construct Validity: The final multivariable-adjusted model included nine predictors: age ≥ 80 [HR = 1.93 (1.29–2.88)], male gender [HR = 1.92 (1.33–2.86)], physical inactivity [HR = 2.26 (1.47–3.49)], use of three or more drugs [HR = 1.52 (1.08–2.14)], sensory deficits [HR = 2.07 (1.21–3.54)], calf circumference <31 cm [HR = 1.91 (1.33–2.75)], IADL disability [HR = 1.89 (1.20–2.96)], gait and balance test ≤ 24 [HR = 1.77 (1.16–2.69)], and pessimism about one’s health [1.70 (1.17–2.48)] [25]. - Criterion Validity & Construct Validity: For each one point increase in the overall score, the corresponding HR for mortality was 1.99 (1.82–2.18), P for trend <0.001. For each one point increase, the corresponding OR (95% CI) was as follows: 1.40 (1.12–1.73) for fractures (P for trend = 0.003); 1.48 (1.26–1.77) for hospitalisation (P for trend <0.001); 1.84 (1.57–2.16) for worsening disability (P for trend = 0.001); 2.21 (1.73–2.83) for new disability (P for trend = 0.001) [25]. | HR indicates good predictive validity of the 9-Item Frailty Measure in predicting mortality. OR’s also indicate acceptable predictive validity in predicting fractures and hospitalisation and good predictive validity in predicting worsening and new disability. All ORs and HR within statistically significant ranges. |
| Brief Clinical Instrument to Classify Frailty | Validity:   - Construct Validity: RR (age and sex adjusted) for institutionalisation: A score of 1 on Brief Clinical Instrument to Classify Frailty; RR = 1·7 (95% CI 1·3–2·1); a score of 2 RR = 3·6 (3·1–4·3) and a score of 3 RR = 9·4 (7·7–11·5). RR for death: For a score of 1 RR = 1·2 (1·0–1·4); a score of 2 RR = 2·0 (1·8–2·2); a score of 3 RR = 3·1 (2·7–3·6) [26].   ROC curve analysis with Geriatric Assessment as a reference standard: AUC 0.77 (SE 0.04, 95% CI 0.69–0.84). Sensitivity (54%, 95% CI 43–64), specificity (100%, 95% CI 88–100) and positive predictive value (100%, 95% CI 91–100) and negative predictive value (44%, % CI 33–55) [27]. | Dose-response relationship between grades of frailty and subsequent institutionalisation and death observed. RRs within statistically significant ranges.  Acceptable AUCs of ROCs indicating the instrument can discriminate between frail and non-frail individuals however low sensitivity and negative predictive value’s also observed. |
| Brief Frailty Index | Validity:   - Content & Construct Validity: The final multivariable-adjusted model based on ADL decline included: Poor balance RR 2.36 (95% CI 1.37–4.04)P = 0 .002, abnormal BMI RR =1.78 (95% CI 1.07–2.95) P=0 .026, impaired Trail-Making Test Part B performance RR=2.34 (95% CI 1.28–4.24)P= 0 .005, depressive symptoms RR =1.83 (95% CI 1.07–3.12) P= 0 .027, and living alone RR =2.19 (95% CI 1.26–3.80)P0 .005 [28]. - Construct Validity: AUC of ROC of Brief Frailty index = 0.76 (95% CI 0.66–0.84). A score of ≥ 3 (vs none) resulted in RR for increased disability = 10.4 (95% CI 4.4–24.2) and RR decreased HRQL =4.2(95% CI =2.3–7.4) after 1 year [28]. | Acceptable AUC of ROC indicating the instrument can discriminate between frail and non-frail individuals. RRs indicate good predictive validity for increased disability and deceased HRQL. |
| British Frailty Index | Validity:   - Construct Validity: EFA and CFA completed; General Specific frailty model fit indices: RMSEA = 0.027, CFI = 0.957 and TLI = 0.964 [29].   Age adjusted HR for mortality per unit increase in frailty scores; 1.7(95% C.I 1.6 - 1.7). Fully adjusted HR = 1.4(1.3-1.5). P values < 0.001 .Fully adjusted HR for hospitalisation per unit increase in frailty score = 1.5(95% C.I 1.4 -1.6) P value < 0.001. Fully adjusted HR for institutionalization =1.6 (95% C.I 1.4 -1.8) P value < 0.001 [29]. | RMSEA, CFI and TLI all within cut off criteria for a good fit. HR’s indicate acceptable predictive validity of the British Frailty Index in predicting mortality, hospitalisation and institutionalization. HR scores within statistically significant ranges. |
| Care Partners-Frailty Index-Comprehensive Geriatric Assessment (CP-FI-CGA) | Validity:   - Construct Validity: RR of morality: 2.15 (95% CI 0.86–5.4) for those with a CP-FI-CGA from 0.3 to 0.5 and 3.87 (95% CI 1.6–9.35) for those with a CP-FI-CGA > 0.5. HR for mortality of 1.04 (95% CI 1.02–1.06) adjusting for age (HR 1.02, 95% CI 0.97–1.07), setting (HR 1.63, 95% CI 0.86–3.1) and gender (HR 2.78, 95% 95% CI 1.54–5.02) for each 1% increment. ROC analysis: AUC 0.71 (95% CI 0.622–0.79) [30].      - Construct & Criterion Validity: Correlation between CP-FI-CGA and FI-CGA r=0.7, P<0.05 [30]. | RR indicates good predictive validity of CP-FI-CGA for scores of> 0.5 in predicting mortality. 95% CI outside of acceptable limits for RR of CP-FI-CGA for scores of 0.3 – 0.5 in predicting mortality.  HR that each 0.01 increase in the CP-FI –CGA was associated with a higher risk of death.  Moderate correlation between CP-FI-CGA and FI-CGA observed. |
| Clinical Frailty Scale | Reliability:   - Inter-rater Reliability: ICC = 0.97, P < 0.001 [32].   Validity   - Construct Validity: Correlation between Clinical Frailty Scale and a Frailty Index: Pearson coefficient 0.80, P < 0.01 [31].   HR for death = 1.30 (CI 1.27–1.33), HR for institutionalisation = 1.46 (1.39–1.53) [31]. In multivariable models adjusted for age, sex and education, each 1-category increment of Clinical Frailty Scale increased the risk of death at 70 months (21.2%, 95% CI 12.5%–30.6%) and entry into institutional care at 70 months (23.9%, 95% CI 8.8%– 41.2%) [31].  Cox Regression Analyses for Time until Death; regression coefficient= 0.230, adjusted HR =1.258, standard error 0.050, P value <0.001, 95% CI 1.159 - 1.357 [32].  Multivariate models (adjusted for age, sex and education) in predicting cognitive decline: Regression coefficient for Poisson model in survivors; Mean 0.40 (95% CI 0.28, 0.53). Prediction of mortality: Regression coefficient for multivariate logistic regression; beta 0.54 (SE: 0.05); OR 1.72 [33].   - Criterion Validity: ROC curve analysis (end point 70 months) for mortality; AUC = 0.70 and entry into an institution; AUC = 0.75 [31].   CHSA Clinical Frailty Scale correlation with Frailty Index; 0.71 (P value <0.001), with age; 0.19 (P value <0.001), 3MS; -0.43 (P value <0.001) and disability; -0.53 (P value <0.001) [32]. | Excellent ICC value however the tests were not blinded.  Pearson’s coefficient indicates a high degree of correlation between Clinical frailty Scale and Frailty Index.  HR scores indicate acceptable predictive validity of the Clinical Frailty Scale in predicting mortality, hospitalisation and institutionalization. HRs within statistically significant ranges.  Acceptable AUCs of ROCs indicating moderate predictive validity for mortality and institutionalisation. |
| Clinical Global Impression of Change in Physical Frailty (CGIC-PF) | Reliability:   - Inter-rater Reliability: Kendall’s multiple-rater concordance coefficient; average agreement rates among 26 physicians were 0.97 for intrinsic frailty alone and 0.98 for all areas of frailty [34]. | High level of Inter-Rater agreement indicated within accepted limits. However Inter-Rater Reliability was assessed through web based scenarios only. |
| Comprehensive Assessment of Frailty (CAF) | Validity:   - Construct Validity: ROC curve analysis, 30 day mortality prediction: AUC 0.71. Correlation between Frailty score and observed 30-day mortality (p < 0.05). Spearman’s correlation between the CAF and EuroSCORE (p = 0.35) and to the STS score (p = 0.42) [35].   Predictive value of CAF-score for one-year mortality (multivariate logistic regression) P-value = 0.001, OR = 1.097, 95% CI = 1.038–1.160. ROC curve analysis; AUC = 0.70 (95% CI 0.60–0.80) [36].  Mann–Whitney test indicated CAFs ability to predict 30-day and 1-year mortality (P ≤ 0.001). CAF prediction of 30-day mortality; OR = 1.1 (95% CI: 1.06–1.2) P = <0.001. 1-year mortality OR = 1.1 (95% CI: 1.06–1.1). Bivariate logistic regression for 1-year mortality prediction by CAF; OR = 1.09 (95% CI: 1.05–1.13; P < 0.001) [37]. | Low level of correlation between CAF & EuroSCORE. AUCs of ROCs just within acceptable range indicating moderate predictive validity for 30 day and 1 year mortality. OR for prediction of 1 year mortality also just within acceptable range. |
| Continuous Composite Measure of Frailty | Validity:   - Construct Validity: Frailty was positively related to age (r = 0.33, p<0.001). Proportional hazards model controlling for age, gender and education: Risk for each 1-unit increase in Continuous Composite Measure of Frailty Score of death; HR: 1.84 (95% CI: 1.28 -2.66), disability; HR: 2.10 (95% CI: 1.56 - 2.81) and IADL disability; HR 1.76 (95% CI: 1.30- 2.40) [38]. - Criterion Validity: Spearman correlations between the Continuous Composite Measure of Frailty and an amended version of the Frailty Phenotype measure; (rho = 0.44, p<0.001) [38]. - Responsiveness: Proportional hazards model controlling for age,   sex, education and baseline frailty; the relationship of change in the rate of frailty and risk of death explored. The risk of death with each 1-unit increase in baseline frailty; HR: 2.29 (95% CI: 1.58, 3.32). The risk of death with each 1- unit increase in annual change in frailty; HR: 4.97 (95% CI: 3.08, 8.02) [38]. | HR scores indicate good predictive validity of the Continuous Composite Measure of Frailty in predicting mortality and disability.  Spearman’s rho indicates a weak relationship between Continuous Composite Measure of Frailty and an amended version of the Frailty Phenotype measure. |
| EASY-Care Two-step Older persons Screening (Easycare TOS) | Reliability:   - Inter-rater Reliability: 89% Inter-rater agreement; Cohen’s Kappa = 0.63 [39].   Validity:   - Construct Validity: Correlation Coefficients calculated between EASY-Care TOS and multimorbidity (0.50), disability (0.53), and mobility (0.55) and moderately with polypharmacy (0.34), cognition (0.31), mental well-being (0.38), and self-perceived health (0.35). All P values < 0.001 [39]. - Criterion Validity: The correlation between EASY-Care TOS and modified Phenotype of Frailty was 0.52, and 0.63 between EASY-Care TOS and a Frailty Index. All P values < 0.001 [39]. | Cohen’s Kappa indicates moderate Inter-rater agreement.  Correlation Coefficients calculated between EASY-Care TOS and related constructs (multimorbidity, disability and mobility) were moderate. Correlations with polypharmacy, cognition, mental wellbeing and self-perceived health were weak.  Correlations observed between EASY-Care TOS and alternate frailty assessment tools indicated a moderate agreement. |
| Edmonton Frail Scale (EFS) | Reliability:   - Internal Consistency: Cronbach’s α = 0.62 [41]. - Inter-rater Reliability: Cohan’s Kappa; k = 0.77, P = 0.0001 (n=18) [41].   Validity:   - Construct Validity: Pearson’s Correlation Coefficient between EFS and Geriatrician’s clinical impression of frailty: 0.64 (P = <0.001), medication: 0.34 (P = <0.001), age: 0.27 (P = 0.015) and sex: 0.05 (P= 0.647). Construct validation of sub-samples, the correlation of EFS with Barthel Index: r = –0.58, P = 0.006, n = 21. Correlation with the MMSE: r = –0.05, P = 0.801, n = 30 [41].   Bivariate analysis: association between frailty according to EFS and LOS (hospital); rho- -0.13 P=0.24. Association between frailty according to EFS and discharge destination; r = -1.32, P=0.19. Association between frailty according to EFS and raw change on the EMS following physiotherapy input; rho -0.06, P = 0.61 and rate of change on the EMS; r= -0.001, P=0.98. OR of achieving a satisfactory level of physiotherapy engagement: 1.43, P=0.02 [41].  EFS scores and LOS and mortality compared: EFS 0-3: mean LOS 7.0 days; EFS 4-6: mean LOS 9.7 days; and EFS ≥7: mean LOS 12.7 days; P= 0.03. Crude mortality rates at 1 year were 1.6% for EFS 0-3, 7.7% for EFS 4-6, and 12.7% for EFS ≥7 (P = 0.05). After adjusting for baseline risk differences using a “burden of illness” score, the HR for mortality for EFS score of 7 compared with EFS score of 0-3 was 3.49 (95% confidence interval [CI], 1.08-7.61; P = 0.002) [43]. | Crohnbach α within an acceptable range indicating acceptable level of internal consistency.  Cohen’s Kappa indicates high inter-rater agreement.  Significant correlation between EFS and Barthel Index observed. Correlation with MMSE not significant.  The use of EFS to assess frailty in a sub-acute hospital cohort was not supported due to poor construct/predictive validity. Spearman’s rho indicates a weak relationship between EFS scores and LOS, institutionalisation and physical functioning.  HR scores indicate good predictive validity of the EFS in predicting mortality in alternate study. |
| Evaluative Index for Physical Frailty | Reliability:   - Inter-rater Reliability: Cohen’s Kappa: 0.72, ICC = 0.96 (n=24) [44]. - Intra-rater Reliability: Cohen’s Kappa: 0.77 and 0.80, ICC = 0.93 and 0.98 (n=24) [44].   Validity:   - Content Validity: 80% agreement on items reached after the third round of Delphi Study to create the definite EFIP [44]. - Construct Validity: Correlation between EFIP and TUG = 0.61, EFIP and POMA = - 0.71 and EFIP and CIRS-G = 0.66. All P values = 0.00 [44]. | Cohen Kappa’s within moderate to high ranges and ICC’s within a good range indicating good inter-rater and intra-rater reliability.  Fair – moderate correlations with TUG, POMA, and CIRS-G. |
| Frailty Index-Comprehensive Geriatric assessment (FI-CGA) | Reliability:   - Inter-rater Reliability: Assessed at baseline and three month follow up; 0.95 and 0.96 respectively [45].   Validity:   - Construct Validity: The unadjusted HRs for adverse outcome (compared with mild frailty) of moderate and severe frailty were 1.9 (95% CI 1.7–2.1) and 5.5 (95% CI 3.6–7.4), respectively [45]. In an alternate study for each increment of frailty measured by the FI-CGA the adjusted HR for death: 1.23 (CI 1.18 -1.29) and for institutionalisation: 1.20 (CI 1.10 – 1.32) [46].   The FI-CGA was notionally correlated (r = 0.33) with the MMSE and moderately correlated (r =~0.55) with measures of function and the comorbidity index (r = 0.57) [45].  Risk of one-month and one-year all-cause mortality calculated by ROC analysis: At one month; AUC =0.724, P <0.0001. For one year; AUC = 0.727, p<0.0001 [47].   - Criterion Validity: Correlation between FI-CGA and a Frailty Index; r = 0.76 [46]. | ICCs in a good range indicating a high level of inter-rater reliability.  HR scores indicate good predictive validity of the FI-CGA in predicting mortality and institutionalisation.  ROC AUCs within acceptable range indicating good predictive validity for 30 day and 1 year mortality.  Moderate correlations between FI-CGA and Frailty Index. |
| Frailty predicts death One yeaR after CArdiac Surgery Test (FORECAST) | Validity:   - Construct Validity: Prediction of 1-year mortality by FORECAST; ROC analysis: AUC 0.76; 95% CI: 0.67– 0.85 [35].   Prediction of 30 day mortality by FORECAST calculated by Logistic regression; OR 1.10 (95% CI: 1.03–1.10; P-value <0.001). Bivariate logistic regression showed that FORECAST is associated with 1-year mortality independently of  Age; OR 1.26 (95% CI: 1.14–1.40; P < 0.001) [36]. | ROC AUCs within acceptable range indicating good predictive validity for 1-year mortality.  ORs indicate acceptable predictive validity in predicting 1-year mortality. ORs within statistically significant ranges. |
| Frailty Index | Validity:   - Construct Validity: In Cox regression analysis; frailty strongly inversely correlated with time to death (r = -0.98, P < 0.01). The average value of the frailty index increased with age in a log-linear relationship (r =0.91; P < 0.001) [48]. | Correlation coefficient’s within high ranges indicating a strong dose response relationship of frailty scores in predicting mortality. |
| Frailty Index based on Primary Care Data. | Validity:   - Content Validity: Adjusted HR: A one deficit increase in the FI score was associated with an increased HR for adverse health outcomes; HR: 1.166; 95% CI 1.129–1.210) and moderate predictive ability for adverse health outcomes (c-statistic: 0.702; 95% CI 0.680–0.724) [50]. - Criterion Validity: FI based on Primary Care Data and GFI; Pearson’s correlation coefficient = 0.544, p-value < 0.001. The ROC analysis; prediction that a randomly selected patient from the high-GFI-score group would also have a high FI score (AUC 0.78, 95% CI 0.74 - 0.82) [49]. | HR scores indicate good predictive validity of the FI based on Primary Care Data in predicting adverse health outcomes. C-statistic for predictive validity within accepted range.  Pearson’s Correlation coefficients and ROC showed moderate correlations between the GFI & FI based on Primary Care Data. |
| Frailty Index for Elders (FIFE) | Reliability:   - Internal consistency: KR20 of FiFE: 0.67 in in assisted living facilities setting and 0.39 in home and community based care setting. KR20 for sub-dimensions of FiFE: Functional Activities; 0.54 in assisted living facilities, 0 .35 in home and community based care, Illness Consequences; 0.61 in assisted living facilities, 0.54 in home and community based care, Health Care Use; 0.54 in assisted living facilities, 0.75 in home and community based care [51].   Validity:   - Content & structural validity: Item discrimination range; 0.25 to 0.88 for the assisted living facilities group ranged and 0.18 to 0.83 in the home and community based care group [51]. | Reliability of FIFE as indicated by KR20 is not high, indicating independence of items. A range of high – low discriminatory values observed. |
| Frail Non-  Disabled Instrument (FiND) | Validity:   - Construct validity: The FiND questionnaire presented 95% specificity (95%CI 75.1–99.2%) and 76% (95%CI 54.9–90.6%) in the identification of nondisabled frail participants [52]. - Construct Validity & Criterion: Agreement between FiND and Frailty Phenotype criteria; kappa = 0.748, weighted kappa = 0.836 (P values = <0.001). Agreement between results of the FiND disability domain and the 400-meter walk test; kappa = 0.920, P = <0.001 [52]. | Good specificity indicated. High level of agreement between FiND & Phenotype of Frailty criteria observed. |
| Frailty Screening Tool | Validity:   - Content & Construct Validity: Multiple logistic regression analysis utilised to identify items associated with frailty: Timed walk; two-tail P value: 0.000, OR: 3.282 (95% CI 1.786–6.030), Pulse pressure; two-tail P value 0.016, OR:2.074 (95%CI 1.144–3.761), Cognitive change; two-tail P value 0.002, OR: 2.641 (95% CI 1.419–4.915), Hearing deficit; two-tail P value =0.011, OR:2.186 ( 95% CI 1.197–3.995) [53].   The finalised Frailty Screening Tool showed a 93% negative predictive value for a score of 0 (n=55, non-frailty=51) and a 70% positive predictive value for a score of 4 (n=10, frailty=7). The Frailty Screening Tool AUC 0.734 (95%CI, 0.661–0.806) [53]. | Acceptable AUC of ROC indicating moderate predictive validity of tool for adverse outcomes. |
| Groningen Frailty indicator (GFI) | Reliability:   - Internal Consistency: Cronbach α 0.77 [60] and 0.73 [56] KR-20: 0.68 [58].   Internal consistency of sub scales of GFI; Daily Activities: Cronbach’s α = 0.81(95% CI = 0.79-0.83), Psychosocial Functioning: Cronbach’s α = 0.80(95% CI = 0.78-0.82), and Health Problems Cronbach’s α = 0.57 (95% CI = 0.54-0.61) [54].   - Inter-rater Reliability: Assessed by 4 independent raters. 3/4 agreement by raters on 60% of cases, 2/4 agreement on 40% (n = 275) [61].   Validity:   - Construct Validity: Correlations between chronological age and frailty as assessed by GFI; r= 0.32, p <0.001. In step wise regression analysis relation of frailty to self-management abilities; Step 1 –0.42 (P<0.001), Step 2 –0.39 (P<0.001) [59].   Cohen's Kappa coefficients between GFI and TFI = 0.74. The association between the GFI & TFI scores: r = 0.87. The correlation coefficients between frailty as measured by GFI and disability measured by GARS: r = 0.57 [57].  Convergent and discriminant validity; assessed using Spearman Rank  Correlations between GFI and diseases and disorders, case complexity, and health care needs life satisfaction, activities of daily living, quality of life and mental health. Convergent validity scores ranged from 0.45 to 0.61 and discriminant validity scores ranged from 0.08 to 0.50 [58].  GFI frail scores OR (adjusted) 2.62 (95% CI 1.48-4.64) for developing disabilities (compared to the GFI non-frail group). Sensitivity and specificity for development of disabilities observed to be 71% and 63% respectively. Mortality OR (unadjusted) 3.29 (95% CI 1.03-10.47), adjusted OR 1.35 (0.32-5.76). Adjusted and unadjusted ORs for hospitalisation; 1.40 (95% CI 0.84-2.33) 1.33 (95% CI 0.73-2.41) respectively [55].  Mokken item response theory model of monotone homogeneity applied for scale analysis: Daily Activities subscale Hs = 0.84 , Psychosocial Functioning  Subscale Hs = 0.54 and Health Problems subscale Hs = 0.35 [54].  In a gastric cancer cohort (n=180) ORs for mortality calculated in multivariate analyses (adjusted for age, neoadjuvant chemotherapy, type of surgery, tumour stage and ASA classification): 4.0 (95%CI 1.1–14.1), P=0.03 [62].  Construct & Criterion Validity: Correlation analysis between GFI subscales and related measures: GFI Daily Activities subscale and RAND-36 physical functioning scale (r = −0.62). Psychosocial Functioning subscale with HADS (r = 0.67) and the Jong Gierveld loneliness scale (r = 0.67). Health Problems subscale with the general health rating of the EuroQol-5D (r = −0.48), the RAND-36 physical functioning (r = −0.53), the HADS (r = 0.36), and the Jong Gierveld Loneliness Scale (r = 0.37) [54].  GFI’s Sensitivity (76%) and specificity (73%) in assessing for frailty in older adults both with and without cancer [58]. Using Fried’s frailty criteria as a reference standard for ROC analysis AUC = 0.64 for GFI, sensitivity 0.57 and specificity 0.72 [56].  ROC curve analysis in cancer cohort; predictive accuracy of tool calculated with Geriatric Assessment as a reference standard: AUC 0.74, SE 0.05, 95% CI 0.65–0.80), Sensitivity (64%, 95% CI 52-72), Specificity (86%, 95% CI 70–95) and positive predictive value (93%, 95% CI 83–87) and negative predictive value (46%, % CI 34–58) [27].  Criterion Validity: FI based on Primary Care Data and GFI; Pearson’s correlation coefficient = 0.544, p-value < 0.001. The ROC analysis; prediction that a randomly selected patient from the high-GFI-score group would also have a high FI score (AUC 0.78, 95% CI 0.74 - 0.82) [49]. | Cronbach α indicate good Internal consistency however and KR-20 scores slightly lower than acceptable range. Internal consistency for daily activities and psychosocial functioning sub scales good. Poor internal consistency for health problems subscale.  Cohen's Kappa coefficient showed a good level of agreement between GFI and TFI scores.  Frailty assessed by GFI positively correlated to age and negatively related to self-management abilities.  Convergent and divergent validity - higher correlations between the GFI observed and  related concepts. With the exception of life satisfaction, lower correlations were  found between the GFI and different concepts.  Adjusted OR for developing disabilities within significant ranges indicating a good level of predictive validity. Unadjusted OR for mortality statistically significant however adjusted OR not. Adjusted and unadjusted ORs for hospitalisation also not significant. In alternate study adjusted OR indicated strong predictive validity for mortality.  Mokken scale analyses scaling coefficients within significant range for daily activities and psychosocial functioning subscales only.  Convergent validity indicated strong to moderate correlations of GFI subscales scores to other assessment tool scores theoretically predicted to correlate with.  Sensitivity and specificity within acceptable ranges.  Moderate correlations observed between GFI and alternate frailty assessment tools. |
| Guilley Frailty Instrument | Validity:   - Construct Validity: Deficiencies in ≥4 of the domains of the Frailty Instrument; RR of ADL dependence = 8.05 (CI 2.69 to 24.08) P < .001. Deficiencies in ≥4 of the domains of the Frailty Instrument; RR of Death = 5.68 (CI 2.37 to 13.62) P < 0.001 [63]. | RR scores indicate good predicative validity of Guilley Frailty Instrument scores in predicting ADL dependency and mortality. |
| Inactivity and Weight Loss | Validity:   - Construct validity: Age-adjusted three year relative risks; increased self-reported disabilities OR 5.2 (95% CI 1.04–25.8), decline in performance tests OR 3.7 (95% CI 0.8–16.2), and mortality OR 4.1 (95% CI 1.8–9.4) [64].   Compared to a weight stable active reference group participants who were inactive and weight loosing reported significantly more diseases (mean 2.2 ±1.7 vs 1.1 ±1.1), disabilities (81% vs 43%), use of medications (mean 2.3 ±1.8 vs 1.5 ±1.5, use of care services (26% vs 6%), lower self-rated health (2.8 ±1.0 vs 3.8 ±0.7), relative health (1.9 ±0.8 vs 2.6 ±0.6) and physical performance score (17% vs 22%). P = 0.001 for all variables [65]. | OR scores indicate good predictive validity in predicting increased self-reported disability, decline in performance tests and mortality. |
| INTER - FRAIL Study Questionnaire | Validity:   - Construct validity: ROC analysis for ability to predict frailty: AUC = 0.695, Sensitivity = 71%, Specificity = 58%.ROC analysis for ability to predict disability: AUC = 0.716, Sensitivity =74%, Specificity = 65% [66]. | ROC AUC’s in acceptable ranges indicating good predictive validity in predicting disability and frailty. Fair sensitivity for both disability and frailty prediction. |
| KLoSHA Frailty Index | Validity:   - Construct Validity: Unadjusted Cox proportional hazard analysis for mortality prediction: HR 7.23 (95% CI 4.01 – 13.05), fully adjusted Cox proportional hazard analysis HR 2.18 (95% CI 1.07–4.45). Harrell’s c-index calculated to establish discrimination for mortality. C-index = 0.713 (95% CI 0.656-0.770). Logistic regression model used to analyse the impact of frailty status on subsequent functional decline (assessed by K-ADL score) and hospitalisation. For functional decline OR pre-frail 4.06 (95% CI 0.42 – 39.99), OR frail 148.00 (95% CI 18.54–1181.72). For hospitalisation: Odds ratios pre-frail 0.90 (95% CI 0.57–1.41), odds ratio frail 2.13 (95% CI 1.04–4.35) [67].   ROC curves analysis for functional decline: AUC = 0.937.For hospitalisations: AUC = 0.543 [67].   - Criterion Validity: Spearman’s rank correlation coefficient between CHS frailty index and KFI = 0.487 (p˂0.001) and between the SOF index and KFI = -0.003 (p=0.949) [67]. | HR, OR and c-index all in acceptable ranges indicating good predictive validity in predicting mortality, functional decline and hospitalisation. All scores statistically significant.  ROC AUC is in excellent range for functional decline. AUC for hospitalisation was poor indicating limited discriminative ability. |
| Marigliano–Cacciafesta Polypathological Scale (MPS) | Validity:   - Construct Validity: Correlation analysis between the MCPS and other geriatric evaluation scales via Pearson’s test. Statistically significant correlations were observed between the MCPS and nutritional state measured by MNA (P < 0.01, r = -0.339), motor functionality measured by the Tinetti Test (P < 0.01, r =-0.388), ADL functioning measured by BI (P < 0.01, r = -0.449) and global functional state measured by the GEFI (P < 0.01, r = 0.428) [68]. | Pearson’s test correlations within statistically  significant ranges indicating convergent validity. |
| Phenotype of Frailty | Validity:   - Construct Validity: Prediction (over three years) of incident falls, worsening mobility or ADL disability, hospitalization and death; HR’s ranging from 1.82 to 4.46, unadjusted, and 1.29–2.24, adjusted. ORs for incident frailty following assessment as pre-frail at baseline = 4.51 (CI 3.39 -6.00) unadjusted and 2.63 (CI 1.94 - 3.56) adjusted for covariates compared to those with no frailty criteria at baseline. P-values <0.0001 [13].   In adjusted multivariate analysis, frail women (identified by Phenotype of frailty) were subsequently at increased risk of recurrent falls (OR 1.38, 95% CI 1.02 1.88), hip fracture (HR 1.40, 95% CI 1.03–1.90), any nonspine fracture (HR 1.25, 95% CI 1.05–1.49), and death (HR 1.82, 95% CI 1.56–2.13) [2].  ROC curve analysis in cancer cohort; predictive accuracy of tool calculated with Geriatric Assessment as a reference standard: AUC 0.72, SE 0.05, 95% CI 0.63–0.81), Sensitivity (52%, 95% CI 46–68), Specificity (92%, 95% CI 83–99) and positive predictive value (94%, 95% CI 84–99) and negative predictive value (59%, % CI 48–70) also determined [27].   - Criterion validity: Correlations between the Phenotype of Frailty and Gill Frailty Measure; Spearman = 0.55 (P < 0.001) and kappa = 0.25 (P < 0.001) [69].   Phenotype of Frailty measure and a Deficit Index included into the same cox regression model for RR for mortality at 4 year follow up showed that the Deficit Index (RR=1.035, CI=1.026-1.045) significantly better predicted death than the Phenotype of Frailty (RR=1..014; CI=1.009-1.019) [70]. | HR and OR in acceptable ranges indicating good predictive validity in predicting incident falls, worsening mobility or ADL disability, hospitalization, and mortality. All scores statistically significant.  Acceptable AUCs of ROCs indicating the instrument can discriminate between frail and non-frail individuals however low sensitivity also observed.  Moderate correlations observed between Phenotype of Frailty and Gill frailty indicator.  Deficit index shown to be a better predictor of death at 4 year follow up than Phenotype of Frailty measure. |
| Predictive Physical Frailty Score | Validity:   - Construct Validity: Ability of Predictive Physical Frailty Score to predict functional decline: c-statistic = 0.71 in an independent sample (n=807) and 0.72 in studies original sample (n=545) [71]. | C-statistic within appropriate range to indicate scores predictive validity in predicting functional decline. |
| Prognostic Risk Score | Validity:   - Construct validity: Cox proportional hazards regression models used to identify and select tool items based on prediction of 3 year mortality; age HR 1.44 (95%CI 1.02- 2.04), male sex HR 2.93 (95% CI 1.89 -4.59), living alone HR 1.53 (95% CI 0.99 - 2.38), BMI 18.5 kg/m2 HR 4.09 (95% CI 2.06 - 8.14), cardiovascular disease HR 1.42 (95% CI 0.94 -2.15), elderly mobility score of 20 HR 1.92 (95% CI 1.24 -2.98), number of medicines >2 HR 2.28 (95% CI 1.21 -4.31), impaired motor skills HR 1.47 (95% CI 0.93 -2.32) and process skills HR 1.92 (95% CI 1.12 - 2.98) in activities of daily living [72].   Three year mortality rates across the tools five frailty groups (good – very poor) were 8.0%, 15.9%, 25.9%, 41.5%, and 68.8%, respectively (P<0.001). In ROC analysis the AUC of the Prognostic Risk Score for the prediction of 3 year mortality = 0.78 (95% CI 0.71 to 0.84) [72]. | Acceptable AUCs of ROCs indicating good predictive validity for 3-year mortality. |
| Self-Report Screening Tool for Frailty | Validity:   - Construct Validity: Multiple logistic regression analysis for co-morbidities of frail and pre-frail compared with robust individuals: Frail OR 4.424 (CI 1.982–9.876) P<0.001. Pre-frail OR 1.889 (CI 1.181–3.022) P =0.008 [73].   Association between baseline frailty and incident hospitalisation at one year follow up; Fisher’s exact test P = 0.35, and incident physical limitation at one year follow up; Fisher’s exact test P = 0.67. For Mortality at 3 year follow up Fisher’s exact test = 0.025 [73]. | OR scores indicate good predictive validity in predicting comorbidity.  Fisher’s exact test within significant and acceptable range for prediction of mortality at 3-year follow up. No significant association indicated by Fisher’s exact test between baseline frailty and incident hospitalisation/incident physical limitations at one year follow up. |
| SHARE Frailty Instrument (SHARE FI) | Validity:   - Construct Validity: In females: the DFactor model included 15,578 cases (standard R^2^ = 0.61). All five frailty indicators of the SHARE FI discriminated well (p < 0.001) between the three classes: non-frail (N = 10,420; 66.9%), pre-frail (N = 4,025; 25.8%), and frail (N = 1,133; 7.3%). Relative to the non-frail class, the age-adjusted OR for mortality at 3-year follow up was 2.1 (95% CI 1.4 - 3.0) in the pre-frail and 4.8 (95% CI 3.1 - 7.4) in the frail. In males: 12,783 cases (standard R^2^ = 0.6, all frailty indicators had p < 0.001). Non-frail (N = 10,517; 82.3%), pre-frail (N = 1,871; 14.6%), and frail (N = 395; 3.1%); Age-adjusted OR for mortality: 3.0 (95% CI 2.3 - 4.0) in the pre-frail, 6.9 (95% CI 4.7 - 10.2) in the frail [74].   In Spanish cohort the age adjusted OR for mortality associated with frailty was 3.2 (95% CI 1.0 to 10.2) for women and 8.3 (95% CI 3.1 to 22.1) for men [75].  ROC curve analysis completed to assess mortality prediction of the SHARE Frailty instrument: Women: SHARE-FI-AUC = 0.77; Men: SHARE-FI-AUC = 0.76 [77].  Spearman correlation coefficients between SHARE FI and ADL and IADL difficulties; >0.3, P <0.001 [76].  Criterion Validity & Construct Validity: Mortality prediction of SHARE FI and a Frailty Index (FI) compared via ROC AUCs. Women: AUC-FI =0.79, 95% CI 0.75 – 0.82, P < 0.001; AUC-SHARE FI = 0.77, 95% CI 0.73 – 0.81, P < 0.001. Men: AUC-FIx= 0.77, 95% CI 0.74 – 0.79, P < 0.001; AUC-SHARE FI = 0.76, 95% CI 0.74 – 0.79, P < 0.001) [78]. | OR scores indicate good predictive validity of SHARE FI in predicting mortality.  Acceptable AUC of ROC indicating good predictive validity for mortality.  SHARE FI shown to have had significant direct cross-sectional correlations with the number of ADL and IDAL difficulties.  SHARE FI and a FI found to be equally predictive of mortality at 2.4 years (mean) follow up. |
| SHARE FI75+ | Reliability:   - Internal Consistency: DFactor analysis for women: Discrimination between the three classes of frailty by each factor: non-frail (N=1205; 36.2%), prefrail (N=1539; 46.3%) and frail (N=581; 17.5%), all P <0.001. Loadings on the DFactor: Fatigue=0.39 (R2=0.15); Low appetite=0.28 (R2=0.08); Weakness=0.56 (R2=0.34); Observed gait=0.48 (R2=0.26); Frequency of activities=0.65 (R2=0.43) [79]. - DFactor analysis for men: Discrimination between the three classes of frailty by each factor: non-frail (N=1213; 46.9%), prefrail (N=1058; 40.9%) and frail (N=316; 12.2%), all P <0.001. Loadings on the DFactor: Fatigue=0.45 (R2=0.20); Low appetite=0.32 (R2=0.12); Weakness=0.62 (R2=0.38); Observed gait=0.43 (R2=0.22); Frequency of activities=0.60 (R2=0.36) [79].   Validity:   - Content & Construct Validity: For frail diagnosis OR for 2 year mortality (adjusted for baseline age, comorbidity and BADL disability); 2.2 (95% CI 1.2 - 3.8) in women and 4.2 (95% CI 2.6 - 6.8) in men. By wave 4, 49% of frail women (78 of 159) had at least one more limitation with BADL (compared with 18% of non-frail, 125 of 684; p<0.001); in men, these proportions were 39% (26 of 66) and 18% (110 of 621), respectively (p<0.001) [79]. | Adjusted OR scores indicate good predictive validity in predicting disability and mortality |
| SOF Frailty Criteria | Validity:   - Construct Validity: Adjusted OR for frailty and risk of falls (OR 2.01, 95% CI 1.05–3.83, p = 0.035), hospitalization (OR 2.08, 95% CI 1.02–4.24, p = 0.045) and death (OR 3.07, 95% 1.02–4.24, p = 0.045) at one-year follow up [80].   Individuals classified as frail by SOF Frailty criteria had a higher age-adjusted risk of recurrent falls (OR 2.38, CI 1.94-2.92), disability (OR 2.17, CI 1.82-2.58), hip fracture (HR1.79 CI 1.46-2.19), and death (HR 2.37 CI 2.14-2.61) (P<.001 for all models) [81].   - Construct Validity & Criterion Validity: SOF frailty criteria vs CHS index. The ROC AUC comparisons revealed no differences between models in discriminating falls (AUC=0.61 for both models; P=.66), disability (AUC=0.64; P=.23), non-spine fracture (AUC=0.55; P=.80), hip fracture (AUC=0.63; P=.64), or death (AUC=0.72; P=.10) [81]. | Adjusted OR scores indicate good predictive validity in predicting falls, hospitalisations, mortality, disability and hip fractures.  Acceptable AUC of ROC for mortality prediction. AUC of ROC below acceptable ranges indicating lower discriminative abilities for falls, disability, non-spine fractures and fractures. |
| Strawbridge Frailty Measure | Validity:   - Construct Validity: OR for death or institutionalisation at 3-year follow-up: 2.286 (95% CI 0.603-8.665). Agreement between a physician frailty assessment and Strawbridge classification = 67% (kappa=0.294) [83]. | 95% CI of OR indicates that there is a limited evidence for predictive validity of Strawbridge Frailty Measure in predicting death or institutionalisation.  Modest agreement between a physician frailty assessment and Strawbridge classification. |
| The Comprehensive Frailty Assessment Instrument | Reliability:   - Internal Consistency: The Cronbach’s α = 0.812 [84] and 0.759 [85] in two separate studies respectively.   Validity:   - Construct Validity: Confirmatory factor analysis and exploratory factor analysis performed; finial model: RMSEA = 0.032 (90% interval = 0.032-0.033), CFI = 0 .974 and TLI = 0 .970 [84]. - Construct Validity &Criterion Validity: Spearman correlation coefficient between the CFAI and the TFI= 0.590 (P = 0.000). Correlations between the physical (r = 0.560, P = 0.000), psychological (r = 0.502, P = 0.000) and social domains (r =0.553, P = 0.000, r=0.311, P = 0.000 and r=0.390, P = 0.000) of CFAI and TFI was acceptable. Environmental domain showed weak correlations with all other domains [85]. | Cronbach α indicate good Internal consistency.  Good to excellent fit indices shown by factor analysis (RMSEA, CFI, and TLI) indicating good structural validity.  CAFI found to correlate well with TFI. Convergent and divergent validity between tools indicated by Spearman correlation coefficients. |
| The Frailty Trait Scale (FTS) | Validity:   - Construct Validity: HR associated with a score in the highest quartile (versus the lowest quartile); HR 2.3 (95% CI 1.6-3.4) for hospitalization, HR 2.5 (95% CI 1.8-3.6) for mortality and HR 3.0 (95% CI 1.6-5.7) for the combined outcome at 3-year follow up [86]. - Construct Validity & Criterion Validity: FTS ROC analysis completed; mortality AUC 0.781 (95% CI 0.739-0.823), hospitalisation AUC 0.681 (95% CI 0.652-0.710), mortality or hospitalisation AUC 0.700 (95% CI 0.672-0.728). AUC comparison with other frailty scales’ predictive accuracy: FTS predicted hospitalization better than the Phenotype of Frailty definition (0.68 vs 0.66, P = .020), no significant difference for mortality prediction [86]. | HR scores indicate good predictive validity for mortality and hospitalisation.  ROC AUC in acceptable ranges for mortality and mortality or hospitalisation prediction. AUC below accepted level for prediction of hospitalisation. |
| The FRAIL Scale | Validity:   - Construct Validity: The FRAIL scale observed to predict all-cause mortality and disability in a graded manner (P for trend <.05). Women who scored in the highest quintile (4or more on the FRAIL scale, identified as frail) had a HR for death of 4.52 (95% CI 3.69–5.54) and an OR for ADL disability of 6.35 (95% CI = 4.35–9.27) compared with those who scored in the lowest quintile (score of 0, not frail) [87]. | HR and OR scores indicate predictive validity in predicting mortality and ADL disability. |
| Tilburg Frailty Indicator (TFI) | Reliability:   - Internal consistency: Cronbach's α= 0.79 [55] and 0.73 [94] Cronbach’s α = 0.70 for the physical domain, 0.63 for the psychological domain, and 0.34 for the social domain [91]. - Test Retest reliability: Pearson correlation coefficient for 2-week interval = 0.90, after 1 year = 0.79 [91].   Validity:   - Construct Validity: Development of disability: Sensitivity 62 (95%CI 52-71), Specificity 71 (95% CI 65-76), and AUC 66 (95% CI 60-72). Mortality: Sensitivity 67 (95% CI 39-87), Specificity 61 (95% CI 56-65), AUC 64 (95% CI 50-78) Hospitalisation: Sensitivity 53 (95% CI 41-64), Specificity 65 (95% CI 60-70), AUC 60 (95% CI 52-67) [55].   ROC analysis utilised to assess predictive validity of TFI frailty scores: Disability; AUC 0.86 (95% CI 0.81–0.92), Sensitivity 0.84, Specificity 0.76. Receiving personal care AUC 0.85 (95% CI 0.78–0.92), Sensitivity 0.91, Specificity 0.63. Receiving nursing care AUC 0.77 (95% CI 0.69–0.86), Sensitivity 0.87, Specificity 0.61. Receiving informal care AUC 0.74 (95% CI 0.67–0.81), Sensitivity 0.71, Specificity 0.63. Visit GP AUC 0.64 (95% CI 0.52–0.76), Sensitivity 0.60, Specificity 0.59. Hospital admission AUC 0.61 (95% CI 0.51–0.71), Sensitivity 0.63, Specificity 0.59 [91].  OR (adjusted) for development of disabilities: 2.00 (95% CI 1.18-3.57). OR (adjusted) for mortality: 1.05 (95% CI 0.24-4.60), OR (adjusted) for hospitalisation 2.59 (95% CI 1.36-4.90) [55].  Correlation analysis between the frailty domains: 0.42 between the physical and psychological domains, 0.19 between the physical and social, and 0.18 between the psychological and social domains (all P<0.001) [91].  After controlling for background characteristics, the TFI predicted disability and the indicators of health care utilization with statistically significant ORs [93].  Predictions of adverse outcomes at 2 year follow up: ROC analysis; disability AUC: 0.81 (95% CI 0.75–0.87), Sensitivity 0.73, Specificity 0.75. Receiving personal care AUC 0.81 (95% CI .74–0.88), Sensitivity 0.86, Specificity 0.66. Receiving nursing care AUC 0.71 (95% CI 0.61–0.82), Sensitivity 0.70, Specificity 0.63. Receiving informal care AUC 0.75 (95% CI 0.68–0.82), Sensitivity 0.69, Specificity 0.69. Receiving residential care AUC 0.78 (95% CI 0.66–0.89). Sensitivity 0.86, Specificity 0.62. Visit to GP AUC 0.58 (95% CI 0.47–0.70), Sensitivity 0.42, Specificity 0.72. Contact with HPC AUC 0.63 (95% CI 0.52–0.74), Sensitivity 0.43, Specificity 0.82. Hospitalisation AUC 0.60 (95% CI 0.51–0.69), Sensitivity 0.53, Specificity 0.62[92].  Linear regression analyses are carried out to examine which determinants predict frailty scores and scores on each of the frailty domains (physical, psychological, social) separately: Physical frailty; the model without the predictor ‘‘multimorbidity’’ explained 22% of physical frailty. Multimorbidity  explained an additional 12.6% of the variance, which represents a medium effect size. Psychological frailty; about 11% was explained by the life-course determinants. Multimorbidity explained an additional 2.4% of the variance of frailty, representing a small effect size. Social frailty; about 15% of social frailty was explained by the life course determinants. Adding multimorbidity to the model neither increased the fit of the model nor affected the  estimates of the effects of the life-course determinants [90].  Four physical frailty components (physical unhealthy, difficulty in maintaining balance, difficulty in walking and physical tiredness), one psychological frailty  component (feeling down) and one social frailty component (lack of social support) predicted future scores on quality of life domains, even after controlling for background characteristics and diseases [89].   - Cross Cultural Validity: Standardised back translation process: Cronbach’s alpha reliability coefficients of the instrument ranged from 0.68 to 0.72 and item-total correlation ranged from 0.12 to 0.52 [94]. - Construct Validity & Criterion Validity: Cohen's Kappa coefficients between GFI & FTI = 0.74, between SPQ & TFI = 0.25. The correlation coefficients between TFI and disability = r = 0.61(99% CI 0.53-0.68) [57]. | Cronbach α indicate good Internal consistency. Good test-retest reliability.  Statically significant adjusted ORs for development of disabilities and hospitalisation indicating good predictive validity. OR for mortality not statistically significant.  ROC AUCs for development of disabilities, hospitalisation and mortality were low indicating limited discriminative ability.  However in an alternate study AUC’s were in excellent – good ranges for prediction of disability, receiving personal care, receiving nursing care and receiving informal care.  ROC AUCs were also within a good range in another alternate study for disability and receiving personal care, receiving nursing care, receiving informal care and receiving residential care indicating good discriminative abilities.  Good agreement between GFI & TFI, Fair agreement between SPQ & TFI.  Effect of the determinants of frailty differs across frailty domains suggesting that dividing the concept of frailty Into domains is justified. |
| WHIOS Multicomponent Measure | Validity:   - Construct Validity: Baseline frailty as a predictor of; risk of death (HR 1.71, 95% CI 1.48–1.97), hip fracture (HR 1.57, 95% CI 1.11–2.20), ADL disability (OR3.15, 95% CI 2.47–4.02), and hospitalisations (OR 1.95, 95% CI 1.72–2.22) after adjustment for demographic characteristics, health behaviours, disability, and comorbid conditions [95]. | HR and OR scores indicate predictive validity in predicting mortality, hip fracture, ADL disability and hospitalisations. |

HR= Hazzard Ratio, CI = Confidence Interval, OR = Odds Ratio, RR = Relative Risk, IADL = Instrumental Activities of Daily Living, AUC = Area Under Curve, ROC = Receiver Operating Curve, SE = Standard Error, ADL = Activities of Daily Living, HRQL = Health Related Quality of Life. EFA = Exploratory Factor Analysis, CFA = Confirmatory Factor Analysis, ICC = Intra-class Correlation Coefficient, S.E = Standard Error, CFI = Comparative Fit Index, TLI = Tucker Lewis Index, RMSEA= Root Mean Square Error of Approximation, STS = Society of Thoracic Surgeons, EuroSCORE = European system for cardiac operative risk evaluation, EMS = Elderly Mobility Scale, LOS = Length of Stay, TUG= Timed “Up & Go” Test, POMA= Performance-Oriented Mobility Assessment, CIRS-G = Cumulative Illness Rating Scale for Geriatrics. R = Correlation Coefficient, CHS = Cardiovascular Health Study, SOF = Study of Osteoporotic Fracture, BADL = Basic Activities of Daily Living, BI = Barthel Index, GEFI = Global Evaluation Functional Index, GDS = Geriatric Depression Scale, MNA = Mini Nutritional Assessment. CHS index = Cardiovascular Health Study Index, H = Loevinger’s scalability coefficient, KR-20 = Kuder-Richardson Formula 20, BMI = body mass index, GP = General Practitioner, HPC = Health Care Professional.

^a^Statistically significant parameters were defined as follows: HR = 1.0 no relationship, HR = >1.0 indicates and adverse relationship, HR = <1.0 indicates a protective relationship, 95% CI must be considered (p429) [98]. For RR and OR the null value = 1.0, If the 95% CI excludes the null value then P = <0.05 (p273) [98]. Logistic regression OR = 1 no relationship, HR = >1 indicates and adverse relationship, HR = <1 indicates a protective relationship (p421) [98]. In ROC correlation analysis AUC = ≥0.7 considered good indication of discriminative power (p208) [99]. Sensitivity and Specificity = 80% is indicative of good sensitivity/specificity (p340 – 341) [98], Cut off for good fit of CFI & TLI = >0.95, for RMSEA = <0.06 (p27) [100]. Pearson Correlation, r=0 no linear relationship, values closer to -1 = stronger negative relationship, value closer to +1 strong positive relationship (p290) [98]. ICC scores of > 0.7 acceptable (p300) [99], Cronbach’s alpha = between 0.7 – 0.95 acceptable (p300) [99], Cohan’s Kappa = 0.4-0.75 fair – good, ≥0.75 excellent (p121) [99].
