# Supplementary material for: Psychometric properties of multicomponent tools designed to assess frailty in older adults: A systematic review
Source: BMC Geriatr. 2016 Feb 29;16:55. doi: 10.1186/s12877-016-0225-2 (PMC4772336; doi:10.1186/s12877-016-0225-2)
Supplement: Additional file 3: — General Characteristics of Studies. (DOCX 44 kb) [file 12877_2016_225_MOESM3_ESM.docx]

Additional file 3: General Characteristics of Studies.

| **Frailty Assessment Tool** | **Study** | **Design & Data Set** | **Study Population:**  **Total N (% female)**  **Mean age years (S.D)** | **Setting** | **Country** | **Final follow Up (months)** |
| --- | --- | --- | --- | --- | --- | --- |
| 9-Item Frailty Measure | Ravaglia et al. [25] | Prospective cohort population-based study  CSBA | 1007 (55.4)  74.7 (± 7.1) | Community, general older adult population | Italy | 48 |
| Brief Clinical Instrument to Classify Frailty | Rockwood et al. [26] | Prospective cohort study  CSHA | 9008 (-)  Aged: ≥65 | Community, general older adult population | Canada | 60 |
|  | Kenig et al. [27] | Prospective cohort study | 135 (55.5)  75.0 (± 6.6) | Inpatient, cancer diagnosis with solid abdominal tumours  in need of surgery | Poland | N/A |
| Brief Frailty Index | Freiheit et al. [28] | Prospective cohort study  CCCS | 374 (27)  71.0 (± 5.9) | Inpatient, coronary artery disease diagnosis | Canada | 12 |
| British Frailty Index | Kamaruzzaman et al. [29] | Prospective cohort study  BWHHS | 4286 (100)  Age range: 60-79 | Community, female older adult population | UK | median 98.4 (range 4 to 111.6) |
| Care Partners-Frailty Index-Comprehensive Geriatric Assessment (CP-FI-CGA) | Goldstein et al. [30] | Validation study | 203 (62.1)  82.2 (± 5.9) | Emergency medical services/geriatric ambulatory care | Canada | 12 |
| Clinical Frailty Scale | Rockwood et al. [31] | Prospective cohort study  CSHA | 2305(-)  Aged: ≥65 | Community, general older adult population | Canada | 60 |
|  | Rockwood et al. [32] | Prospective cohort study  CSHA | 728 (73.4)  87.0 (± 6.72) | Long-term care institutions | Canada | 60 |
|  | Mitiniski et al. [33] | Prospective cohort study  CSHA | 2,305 (62.1)  83.1 (± 6.9) | Community, general older adult population | Canada | 60 |
| Clinical Global Impression of Change in Physical Frailty (CGIC-PF) | Studenski et al. [34] | Qualitative and quantitative instrument development | 6 expert panel members, 46 clinicians, 24 patients, and 12 caregivers (-)  - | Community | USA | N/A |
| Comprehensive Assessment of Frailty (CAF) | Sundermann et al. [35] | Validation study | 400 (51.5)  80.1 (± 4.0) | Inpatient, population undergoing cardiac surgery | Germany | 1 |
|  | Sundermann et al. [36] | Validation study | 213 (51.6)  80.1 ( ± 4.0) | Inpatient, population undergoing elective and urgent cardiac surgery | Germany | 12 |
|  | Sundermann et al. [37] | Validation study | 450 (49.5)  79.0 (± 4.0) | Inpatient, population undergoing elective cardiac surgery | Germany | 12 |
| Continuous Composite Measure of Frailty | Buchman et al. [38] | Longitudinal study  Rush Memory and Aging Project. | 832 (74.4)  80.4 (± 6.9) | Community, general older adult population | USA | 96 |
| EASY-Care Two-step Older persons Screening (Easycare TOS) | Van Kempen et al. [39] | Validation study | 587 (56)  77.0 (± 6.5) | Community, 6 GP practices | The Netherlands | N/A |
|  | Van Kempen et al. [40] | Observational pilot study | 141 (62.0)  77.0 (±6.0) | Community,7 GP practices | The Netherlands | N/A |
| Edmonton Frail Scale (EFS) | Rolfson et al. [41] | Validation study | 158 (53.0)  80.4 (±6.8) | Inpatient and community, referral for a CGA | Canada | N/A |
|  | Haley et al. [42] | Validation study | 86 (51.2)  81.3 (±7.7) | Inpatient, sub-acute hospital | Australia | 1-2 |
|  | Graham et al. [43] | Pilot cohort study | 183 (31.2)  Aged: ≥ 65 | Inpatient, acute coronary syndrome diagnosis | Canada | 36 |
| Evaluative Index for Physical Frailty (EFIP) | De Vries et al. [44] | Delphi study and observational study | 24 (62.5)  78.0 (±6.9) | Mixed; community dwelling and residential care | The Netherlands | N/A |
| Frailty Index-Comprehensive Geriatric Assessment (FI-CGA) | Jones et al. [45] | Secondary analysis of a 3-month randomized  controlled trial  MGAT | 169 (-)  Aged: ≥65 | Community-dwelling frail elders | Canada | 12 |
|  | Jones et al. [46] | Prospective cohort study  CSHA | 2305 (62.1)  Aged: ≥65 | Community, general older adult population | Canada | 60 |
|  | Pilotto et al. [47] | Prospective cohort study | 2033 (57.0)  79.8 (±7.8) | Inpatient, 20 geriatric wards | Italy | 12 |
| Frailty predicts death One yeaR after CArdiac Surgery Test (FORECAST) | Sundermann et al. [35] | Validation study | 213 (51.6)  80.1 ( ± 4.0) | Inpatient, population undergoing elective and urgent cardiac surgery | Germany | 12 |
|  | Sundermann et al. [36] | Validation study | 450 (49.5)  79.0 ( ± 4.0) | Inpatient, population undergoing elective cardiac surgery | Germany | 12 |
| Frailty Index | Mitnitski et al. [48] | Prospective cohort study  CSHA | 2914 (64.4)  82.0 (± 7.43) | Community, general older adult population | Canada | 60 |
| Frailty Index based on Primary Care Data. | Drubbel et al. [49] | Cross-sectional, observational study | 638 (52.8)  73.4 (± 9.2) | Community, general older adult population | The Netherlands | N/A |
|  | Drubbel et al. [50] | Retrospective cohort study | 1679 (59.0)  73.0 (-) | Community, general older adult population | The Netherlands | 24 |
| Frailty Index for Elders (FIFE) | Tocchi et al. [51] | Cross-sectional design validation study  HRQL-ELTC | 312 (77.6)  Aged: ≥65 | Community, home based care and assisted living facilities | USA | N/A |
| Frail Non-  Disabled Instrument (FiND) | Cesari et al. [52] | Validation study | 45 (62.2)  72.5 (± 8.2) | Community, general older adult population | France | N/A |
| Frailty Screening Tool | Doba et al. [53] | Prospective cohort study | 407 (54.8)  78.0 (±4.0) | Community, recruitment from Life Planning Centre Foundation | Japan | 60 |
| Groningen Frailty indicator (GFI) | Bielderman et al. [54] | Cross-sectional study | 1508 (49.3)  75.0 (±7.0) | Community, general older adult population | The Netherlands | N/A |
|  | Daniels et al. [55] | Longitudinal prospective cohort study | 532 (58.5)  77.2 (±5.5) | Community, general older adult population | The Netherlands | 12 |
|  | Drubbel et al. [49] | Cross-sectional, observational study | 638 (52.8)  73.4 (±9.2) | Community, general older adult population | The Netherlands | N/A |
|  | Hoogendijk et al. [56] | Cross-sectional.  Dutch Identification of Frail Elderly Study | 102 (56.9)  78.6 (±7.1) | Community, primary care | The Netherlands | N/A |
|  | Kenig et al. [27] | Prospective cohort study | 135 (55.5)  75.0 (± 6.6) | Inpatient, cancer diagnosis with solid abdominal tumours  in need of surgery | Poland | N/A |
|  | Metzelthin et al. [57] | Cross-sectional study | 532 (58.5)  77.2 (±5.5) | Community, general population, | The Netherlands | N/A |
|  | Peters et al. [58] | Cross-sectional study | 353 (65.0)  81.0 (±8.0) | Community dwelling and residential care | The Netherlands | N/A |
|  | Schuurmans et al. [59] | Validation study | 1338 (-)  74.2 (±6.59) | Community, general population | The Netherlands | N/A |
|  | Smets et al. [60] | Observational prospective cohort study.  KLIMOP | With cancer:  108 (65.0)  median age 76 (70–88)  Without cancer:  290 (64.0)  median age 78 (70–97) | Inpatient and community, cancer diagnosis | Belgium & The Netherlands | N/A |
|  | Steverink et al. [61] | Validation study | 275 (72.9)  78.0 (±7.0) | Hospital inpatients, nursing home residents and community | The Netherlands | N/A |
|  | Tegels et al. [62] | Validation study | 180 (41.1)  69.8 (-) | Inpatient, gastric adenocarcinoma diagnosis and underwent surgical treatment | The Netherlands | 6 |
| Guilley Frailty Instrument | Guilley et al. [63] | Longitudinal study.  SWILSO-O | 1225 (49.4)  81.9 (-) | Community, octogenarians | Switzerland | 18 |
| Inactivity and Weight Loss | Chin et al. [64] | Validation study.  Zutphen Elderly Study (Longitudinal study). | 450 (0.0)  75.0 (-) | Community, general older adult population | The Netherlands | 36 |
|  | Chin et al. [65] | Validation study.  SENECA (Longitudinal study). | 849 (50.9)  77.0 (-) | Community, non-institutionalised, 9 European countries | Belgium (12%), Denmark (11%), France (10%), Italy (13%), The Netherlands (13%), Portugal (16%), Spain (8%), Switzerland (14%), Poland (2%) | 48 - 60 |
| INTER-FRAIL Study Questionnaire | De Bari et al. [66] | Prospective cohort study | 1037 (-)  Aged:≥70 | Community-based | Italy | 36 (n331) |
| KLoSHA Frailty Index | Jung et al. [67] | Population based prospective cohort study  KLoSHA | 693 (50.8)  75.9 (±8.9) | Community, general older adult population | Korea | 67 |
| Marigliano–Cacciafesta Polypathological Scale (MPS) | Amici et al. [68] | Validation study | 180 (63.8)  79.5 (-) | - | Italy | N/A |
| Phenotype of Frailty | Esrund et al. [2] | Prospective cohort study  SOF | 6724 (100)  76.7(±4.9) | Community, female population | USA | 108 |
|  | Fried et al. [13] | Prospective cohort observational study.  CHS | 5317 (58)  Aged: ≥65 | Community, general older adult population | USA | 84 |
|  | Kenig et al. [27] | Prospective cohort study | 135 (55.5)  75.0 (± 6.6) | Inpatient, cancer diagnosis with solid abdominal tumours  in need of surgery, | Poland | N/A |
|  | Kim et al. [69] | Validation study | 162 (0.0)  83.7 (±6.1) | male veterans, geriatric clinic | USA | N/A |
|  | Kulminski et al. [70] | Longitudinal cohort Study  CHS | 4721 (-)  Aged: ≥65 | Community, general older adult population | USA | 132 |
| Predictive Physical Frailty Score | Carriere et al. [71] | Longitudinal cohort Study  EPIDOS | 545 (100)  Median: 79 (IQR (76–81) | Community, female population | France | 84 |
| Prognostic Risk Score | Pijpers et al. [72] | Prospective cohort observational study | 401 (62.1)  78.0 (±6.5) | Referral to the DOC-PG, psychogeriatric patients (80.8% diagnosed with dementia) | The Netherlands | 36 (Median = 26) |
| Self-Report Screening Tool for Frailty | De Souto Barreto et al. [73] | Validation study | 398 (64.3)  Aged: ≥60 | Community, users of the medical  insurance of the French national education system | France | 36 |
| SHARE Frailty Instrument (SHARE FI) | Romero-Ortuno et al. [74] | Longitudinal, population-based study  SHARE | 31115 (55.6)  Female 63.6 (±11.1)  Male 64.1 (±9.9)  Overall 63.8 | Community, general population aged ≥50 years | Austria, Germany, Sweden, Netherlands, Spain, Italy, France, Denmark, Greece, Switzerland, Belgium and Israel | Mean = 28.8 |
|  | Romero-Ortuno et al. [75] | Longitudinal, population-based study  SHARE – Spanish sample | 2221 (57.6)  65.6 (-) | Community, general population aged ≥50 years | Spain | Mean = 28.8 |
|  | Romero-Ortuno et al. [76] | Longitudinal, population-based study  SHARE | 17567 (-)  63.3 (-) | Community, general population aged ≥50 years | Austria, Germany, Sweden, Netherlands, Spain, Italy, France, Denmark, Greece, Switzerland, Belgium and Israel | Mean = 28.8 |
|  | Romero-Ortuno et al. [77] | Longitudinal, population-based study  SHARE | 28162 (54.8)  - | Community, general population aged ≥50 years | Austria, Germany, Sweden, Netherlands, Spain, Italy, France, Denmark, Greece, Switzerland, Belgium and Israel | Mean = 28.8 |
|  | Romero-Ortuno et al. [78] | Longitudinal, population-based study  SHARE | 28361 (54.9)  - | Community, general population aged ≥50 years | Austria, Germany, Sweden, Netherlands, Spain, Italy, France, Denmark, Greece, Switzerland, Belgium and Israel | Mean = 28.8 |
| SHARE Frailty Instrument 75+ (SHARE-FI75+) | Romero-Ortuno et al. [79] | Longitudinal, population-based study  SHARE | 7058 (56.7)  Female 81.1 (±4.9)  Male 80.4 (±4.6)  Overall 80.7 | Community, general population aged ≥50 years | Austria, Germany, Sweden, Netherlands, Spain, Italy, France, Denmark, Greece, Switzerland, Belgium and Israel | 48 |
| SOF Frailty Criteria | Bilotta et al. [80] | Prospective cohort study | 265 (71.0)  81.5 (±6.8) | Community, referral to geriatric outpatient medicine clinic by GP | Italy | 12 |
|  | Ensrud et al. [81] | Prospective cohort study  SOF | 6701 (100)  76.7 (±4.8) | Community, female population | USA | Mean = 115.2 |
| Strawbridge Frailty Measure | Strawbridge et al. [82] | Longitudinal study  Alameda County study | 574 (57.0)  74.0 (-) | Community, non-institutionalised | USA | 348 |
|  | Matthews et al. [83] | Pilot study | 48 (29)  76.2 (-) | Community, outpatient geriatric practice | USA | 36 |
| The Comprehensive Frailty Assessment Instrument | De Witte et al. [84] | Validation study  BAS | 33,629 (51.0)  70.0 (-) | Community, general older adult population | Belgium | N/A |
|  | De Witte et al. [85] | Validation study | 178 (67.2)  Median: 74.0 | Community | Belgium | N/A |
| The Frailty Trait Scale (FTS) | Garcia-Garcia et al. [86] | Prospective cohort study  TSHA | 1829 (56.1)  75.1 (±5.1) | Community, general older adult population | Spain | Mean = 42 |
| The FRAIL Scale | Lopez et al. [87] | Longitudinal study  ALSWH | 8,646 (100)  77.8 (-) | Community, female population | Australia | Mean = 81.6 |
| Tilburg Frailty Indicator (TFI) | Andreasen et al. [88] | Translation and cultural adaption study | 34 (62)  78.8 (6.9) | Community and inpatients older adult population | Denmark | N/A |
|  | Daniels et al. [55] | Longitudinal prospective cohort study | 532 (58.5)  77.2 (±5.5) | Community, general older adult population | The Netherlands | 12 |
|  | Gobbens & Van Assen. [89] | Longitudinal study | 269 (56.8)  80.2 ( ± 3.8) | Community, general older adult population | The Netherlands | 48 |
|  | Gobbens et al. [90] | Cross-sectional study | 484 (57.2)  80.3 (± 3.8) | Community, general older adult population | The Netherlands | N/A |
|  | Gobbens et al. [91] | Cross-sectional study | Sample 1: 245 (54.7)  80.3(± 3.9)  Sample 2: 234 (59.0)  80.2 (±3.7) | Community, general older adult population | The Netherlands | 12 (n 343) |
|  | Gobbens et al. [92] | Validation study | 484 (56.8)  80.3 (± 3.8) | Community, general older adult population | The Netherlands | 24 (n 266) |
|  | Gobbens et al. [93] | Validation study | 245(54.7)  80.3(± 3.9) | Community, general older adult population | The Netherlands | 24 (n141) |
|  | Metzelthin et al. [57] | Cross-sectional study | 532 (58.5)  77.2 (±5.5) | Community, general older adult population | The Netherlands | N/A |
|  | Uchmanowicz et al. [94] | Cross cultural validation study | 100 (58)  68.2 (±6.5) | Community, Primary Care | Poland | N/A |
| WHIOS Multicomponent Measure (WHI-OS) | Woods et al. [95] | Prospective cohort study | 40657 (100)  Age range: 65-75 | Community, female population | USA | 70.8 |

- = No available information, CBSA = Conselice Study of Brain Ageing, ADL = Activities of daily living, CSHA = Canadian Study of Health and Aging, HRQL = Health related quality of life, CCCS = Calgary Cardiac and Cognition Study , BWHHS = The British Women’s Heart and Health Study, GP = General Practitioner, CGA = Comprehensive Geriatric Assessment, MGAT = Mobile Geriatric Assessment Team trial, HRQL-ELTC = Health Related Quality of Life: Elders in Long-Term Care , KLIMOP = Dutch acronym for project on older cancer patients in Belgium and the Netherlands, SWILSO-O = Swiss Interdisciplinary Longitudinal Study on the Oldest Old, SENECA = Survey in Europe on Nutrition and the Elderly, a Concerted Action, KLoSHA = Korean Longitudinal Study on Health and Aging, CHS = Cardiovascular Health Study, SOF = Study of Osteoporotic Fractures, DOC -PG = Diagnostic Observation Centre for PsychoGeriatric patients, EPIDOS = EPIDemiologie de l’OStéoporose (Epidemiology of Osteoporosis), SHARE = Survey of Health, Ageing and Retirement in Europe, BAS = Belgian Ageing Studies, TSHA = The Toledo Study for Healthy Aging, ALSWH = Australian Longitudinal Study on Women’s Health, WHI-OS = Women’s Health Initiative Observational Study.
